# HIV-1 signalling remodels nuclear pores to licence infection

**DOI:** 10.1038/s41586-026-10453-3

**Published:** 2026-05-06

**Authors:** Dejan Mesner, Matthew V. X. Whelan, Maitreyi Shivkumar, Ann-Kathrin Reuschl, Riccardo Zenezini Chiozzi, Konstantinos Thalassinos, Robertus A. M. de Bruin, Clare Jolly

**Affiliations:** 1 Division of Infection and Immunity, University College London, London, United Kingdom; 2 Institute of Structural and Molecular Biology, Division of Biosciences, University College London, London, United Kingdom; 3 Institute of Structural and Molecular Biology, School of Natural Sciences, Birkbeck College, University of London, London, United Kingdom; 4 Laboratory for Molecular Cell Biology, University College London, London, United Kingdom; 5 Present Address: Centre for Immunobiology and Infection, Blizard Institute, Faculty of Medicine and Dentistry, Queen Mary University of London, London, United Kingdom; 6 Present Address: Leicester School of Pharmacy, De Montfort University, Leicester, United Kingdom

**Keywords:** Virus-host interactions, T cells

## Abstract

HIV-1 is readily detected in resting CD4^+^ T cells in vivo^1–4^. However, resting T cells are highly refractory to cell-free virus infection in vitro^5–7^ and require mitogenic activation to become permissive. This paradox raises the fundamental question of what makes a T cell permissive for HIV-1. Here we address this and show that HIV-1 capsid nuclear import at the nuclear pore complex (NPC) is a bottleneck to resting T cell infection, but that HIV-1 overcomes this by triggering receptor-mediated signalling during cell–cell spread to drive nuclear import and licence infection. Coupling viral and cellular assays with super-resolution imaging, we show that contact between HIV-1 infected and uninfected T cells triggers CD4–LCK signalling that activates CDK1, independent of cell-cycle entry, phosphorylating nucleoporins and priming the NPC to promote HIV-1 nuclear import. Critically, cell–cell contact also accelerates nuclear import in activated T cells, providing a paradigm for why cell–cell spread dominates infection. By contrast, HIV-1 virions do not trigger this response, explaining why resting T cells cannot be efficiently infected by cell-free virus. We propose that HIV-1 has evolved to selectively activate CD4 signalling during cell–cell spread to regulate infection at the step of the NPC, offering an explanation for how resting T cells can be infected in vivo.

## Abstract (web summary)

HIV-1 capsid nuclear import at the nuclear pore complex is a bottleneck to resting T cell infection, but HIV-1 overcomes this by triggering receptor-mediated signalling during cell–cell spread to drive nuclear import and licence infection.

## Main

Resting T cells have potent barriers to cell-free human immunodeficiency virus type-1 (HIV-1) infection in vitro^5–7^. Consequently, mitogenic activation to render primary T cells permissive to infection is a ubiquitous feature of HIV-1 research and has led to the notion that activation state dictates T cell permissivity. However, it remains unclear to what extent infection requires T cell activation in vivo, and whether resting T cells containing integrated provirus reflect direct infection of resting T cells^1–3^ or previous infection of activated T cells that have since returned to a resting state^8^. For successful infection, HIV-1 binds to CD4 on the surface of target cells, followed by co-receptor, CXCR4 or CCR5, leading to virus fusion at the plasma membrane and release of capsid into the cytoplasm^9^. The capsid core containing the viral genome traffics to the nuclear envelope (NE) and translocates through the NPC^10,11^ into the nucleus, where the reverse-transcribed viral DNA integrates into the host chromatin. Critically, understanding how HIV-1 achieves this in primary T cells and what makes a T cell susceptible to infection is confounded by the use of in vitro activation, which triggers substantial changes in cell biology, including widespread activation of signalling pathways, breakdown of the NE initiated by NPC disassembly and mitotic cell division^12^.

Lymphoid tissue in people with HIV-1 contains a mix of infected and uninfected CD4^+^ T cells and is an environment in which T cells are densely packed and frequently interact. HIV-1 exploits this to rapidly and efficiently disseminate by cell–cell spread (CCS) at virus-induced immune cell contacts termed virological synapses (VSs)^13^. CCS requires the HIV-1 envelope glycoprotein (Env) expressed on the surface of an infected (donor) T cell to engage CD4 on the surface of an uninfected (target) T cell^13^. This drives sustained cell–cell contact and polarizes HIV-1 budding, leading to highly efficient target T cell infection^13–16^. CCS dominates infection^13–16^; this is attributed to a higher multiplicity of infection (MOI) after the release of large viral loads directed towards uninfected target T cells at the contact zone and short diffusion distances. Our work previously challenged the notion that large viral loads mediated by CCS explain increased infection by demonstrating that exposure of resting T cells to a high dose of cell-free HIV-1 does not result in efficient infection^16^.

Here we demonstrate that, in resting T cells, nuclear import (NI) of HIV-1 capsid is a rate-limiting barrier to infection, but this is overcome by CCS-driven CD4–LCK signalling and CDK1 activation that enhances capsid NI by remodelling the NPC to drive infection. Our results explain how resting T cells can be infected by HIV-1 through CCS and identify a mechanism of HIV-1 regulation of the NPC during natural infection of primary human T cells.

## Cell contact triggers T cell permissivity

**Fig. 1:**
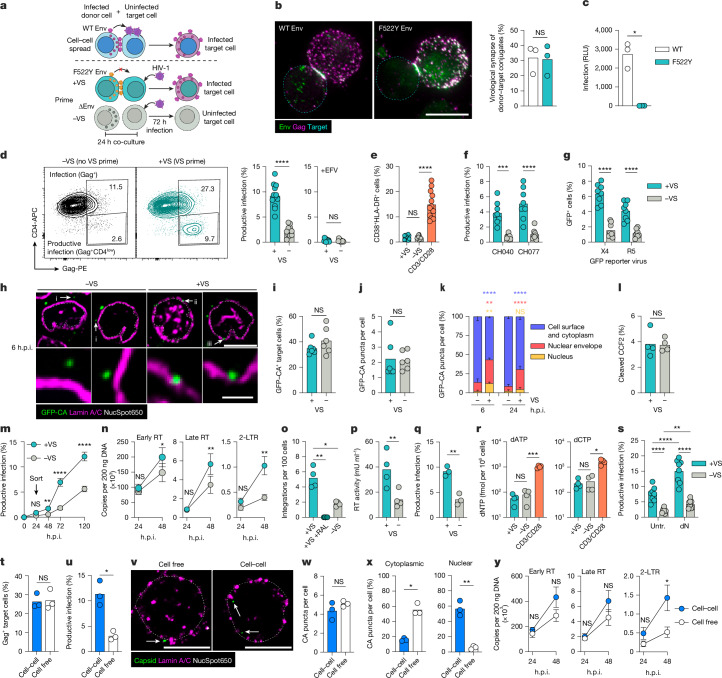
VS formation drives resting T cell permissivity to HIV-1 by enhancing NI. **a**, Schematic. Donor cells infected with HIV-1 WT, Env-F522Y or ΔEnv were co-cultured with resting target cells for CCS or with or without VS-priming. **b**, VS formation by WT or Env-F522Y with quantification; *n* = 50 conjugates per donor. Scale bar, 5 µm. **c**, CCS of the indicated viruses. RLU, relative light units. *n* = 3. **d**, VS-priming increases target infection. Representative flow cytometry plots and quantification of productive infection (Gag^+^CD4^low^) with or without EFV. *n* = 12. **e**, VS-primed targets retain resting state. *n* = 12. **f**,**g**, Infection with transmitter–founder isolates (**f**) and CXCR4-tropic (X4) or CCR5-tropic (R5) GFP-reporter viruses (**g**) with or without VS-priming. *n* = 8. **h**–**k**, GFP–CA virus in targets with or without VS-priming was analysed using iSIM. *n* = 6. **h**, Representative image of GFP–CA localizing to (i) cell surface/cytoplasm, (ii) NE and (iii) nucleus. Scale bars, 5 µm (top) and 1 µm (bottom). **i**, GFP–CA^+^ targets. **j**, Average GFP–CA puncta per GFP^+^ cell. **k**, The distribution of cellular GFP–CA puncta. Statistical comparisons are colour coded. **l**, Viral fusion is VS-prime independent (cleaved CCF2). *n* = 4. **m**–**r**, Target cells were sorted 24 h after priming. *n* = 4. **m**,**n**, Quantification of productive infection (**m**) and early/late RT-products and 2-LTR circles (**n**). **o**,**p**, Proviral integration (**o**) and virus release (**p**) at 72 h.p.i. **q**, Virus spread to secondary/fresh targets at 48 h. *n* = 3. **r**, dATP and dCTP quantification in sorted targets at 48 h.p.i. *n* = 4. **s**, Infection of deoxynucleoside-pretreated (dN) (24 h) or untreated (untr.) cells. *n* = 8. **t**–**y**, Comparison of cell–cell and cell-free infection. *n* = 3. **t**, Gag^+^ targets at 6 h.p.i. **u**, Productive infection at 72 h.p.i. **v**, Intracellular localization of capsid by iSIM at 6 h.p.i. Scale bar, 5 µm. **w**, The average number of CA puncta per cell. **x**, Subcellular CA localization. **y**, Early/late RT products and 2-LTR circles in sorted targets. *n* = 3. Unless stated differently, target cells were infected for 72 h. Data are mean ± s.e.m. or individual values shown from independent donors. Statistical analysis was performed using paired two-tailed *t*-tests (**b**–**d**, **h**–**k**, **p**–**q** and **t**–**x**), one-way analysis of variance (ANOVA) with Dunnett’s post test (**e**, **o** and **r**) and two-way ANOVA with Šídák’s post test (**f**, **g**, **l**–**n**,** s** and **y**). NS, not significant; *P < 0.05, **P < 0.01, ***P < 0.001, ****P < 0.0001. The diagram in **a** was created using BioRender; Mesner, D. https://BioRender.com/ghqita5 (2026). Source data

**Extended Data Fig. 1:**
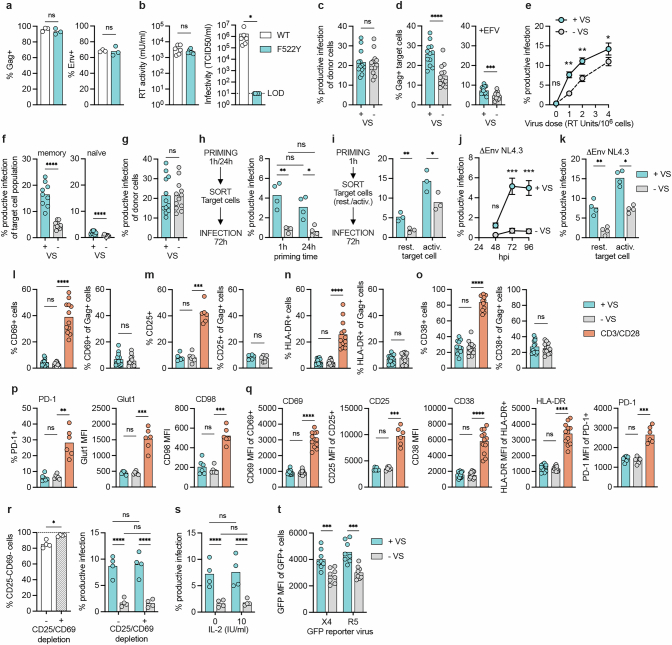
Related to Fig. 1. (**a**) Intracellular Gag and surface Env in Jurkat T cells infected (48hpi) with WT or F522Y Env HIV-1 (VSVg-pseudotyped), n = 3. (**b**) Virus release from HEK293T cells (RT activity) ans supernatant infectivity (TCID50/ml), n = 6. LOD, limit of detection. (**c**) Donor cell infection (48hpi, at the time of target cell addition, from Fig. 1d, n = 12. (**d**) Infection of target cells (Gag + , 72hpi) from Fig. 1d, n = 12. (**e**) Productive infection of resting targets infected with increasing virus doses, n = 6. (**f**) Productive infection of resting memory or naïve targets, n = 9. (**g**) Productive infection of donor cells at 72 h post co-culture with target cells, n = 12. (**h**) Resting target cells were primed (+/− VS) for 1 h or 24 h, then flow sorted from donor cells and infected for 72 h, n = 4. (**i**) Resting (rest.) or activated (active.) target cells were primed (+/− VS) for 1 h, then flow sorted from donor cells and infected for 72 h, n = 3. (**j**) Productive infection of resting target cells infected with Env-pseudotyped ΔEnv NL4.3 virus, n = 4. (**k**) Productive infection of resting (rest.) or activated (active.) target cells infected with Env-pseudotyped ΔEnv NL4.3 virus at 72 hpi, n = 4. (**l-o**) Percentage expression of CD69, CD25, HLA-DR and CD38 on uninfected Gag- (left panels) or infected Gag+ (right panels) target T-cells at 72 hpi, or after 72 h CD3/CD28 stimulation, n = 6. (**p**) Expression of PD-1, Glut1 and CD98 on target T-cells at 72hpi, or after 72h CD3/CD28 stimulation, n = 6. (**q**) Expression (MFI) of CD69, CD25, CD38, HLA-DR and PD-1 in marker-positive population in cells from (l-p). (**r**) CD25 + /CD69+ cells were depleted during CD4 T-cell isolation. Shown is efficiency of depletion (left panel) and infection at 72hpi (right panel), n = 4. (**s**) Infection of target cells in presence or absence of IL-2 in culture medium, n = 4. (**t**) GFP MFI of GFP+ targets from Fig. 1g, n = 8. Mean ± SEM or individual values from independent primary cell donors are shown. Unless otherwise stated target T-cells were infected with HIV-1 NL4.3 for 72 h. a, c-d,f-g, l-o, r) two-tailed paired t-test; b) two-tailed Wilcoxon test; l-q) one-way ANOVA with Dunnett post-test; e, h-k, r-t) two-way ANOVA with Šídák post-test. ns, non-significant; *, *p* < 0.05; **, *p* < 0.01, ***, *p* < 0.001; ****, *p* < 0.0001. Source data

**Extended Data Fig. 2:**
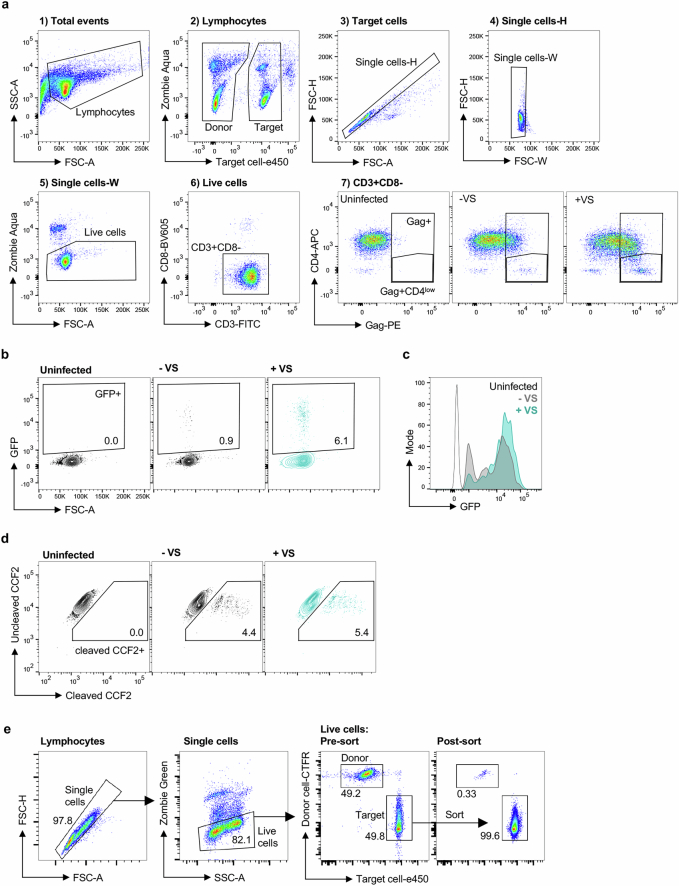
Flow cytometry gating strategy. (**a**) Gating strategy to identify 1) Lymphocytes (FSC-A vs SSC-A), 2) target T-cells (target T-cell dye e450 vs Zombie Aqua dye), 3) single cells-H (FCS-A vs FSC-H), 4) single cells-W (FSC-W vs FCS-H), 5) live cells (FSC-A vs Zombie Aqua dye), 6) CD3 + CD8- cells (CD3 vs CD8), 7) Gag+ (infected) and Gag+CD4^low^ (productively infected) cells (Gag vs CD4). This gating strategy relates to all flow cytometry data in the figures and Extended data. (**b**) Representative contour plots showing gating of GFP+ target T-cells in CD3 + CD8- population from Fig. 1g. Percentages are shown. (**c**) Representative histogram showing GFP expression in GFP+ population (− VS and + VS) or total uninfected target T-cell population from b). (**d**) Representative contour plot showing CCF2 dye cleavage in Blam-Vpr assay from Fig. 1l. **e)** Gating strategy to sort live target cells. Related to Fig. 1m–r and Extended Data Fig. 1h–I and Fig. 4. FSC, forward scatter; SSC, side scatter; -A, area; -H, height; -W, width.

We first established an assay to uncouple the process of Env-dependent cell–cell contact (VS formation) from concomitant virus transfer during CCS, separating the effect of contact-induced stimulation of resting T cells from high MOI during CCS (Fig. 1a). To do this, we mutated the Env fusion peptide (Env-F522Y) in full-length HIV-1 NL4.3. This renders virions transferred across the VS non-infectious by preventing fusion and entry, while maintaining the ability of Env expressed on infected T cells to bind to CD4 and co-receptors on target T cells during cell–cell contact^17^ (Fig. 1b,c and Extended Data Fig. 1a,b). To infect donor T cells with Env-F522Y HIV-1, we pseudotyped virions with wild-type (WT) primary isolate Env. Donor T cells express Env-F522Y on their surface (Extended Data Fig. 1a) and form VSs equivalent to WT HIV-1 (Fig. 1b); however, there is no infection of target T cells by CCS (Fig. 1c). We term this VS-priming. Next, activated primary CD4^+^ T cells (donor cells) were infected with pseudotyped Env-F522Y HIV-1 for 48 h (Extended Data Fig. 1c) and co-cultured with autologous resting primary CD4^+^ T cells (target T cells) for 24 h, enabling cell–cell contact and VS-priming (+VS). Alternatively, resting primary target T cells were co-cultured with activated donor CD4^+^ T cells infected with HIV-1 that does not express Env (ΔEnv), such that infected donors are incapable of VS formation (−VS) (Fig. 1a and Extended Data Fig. 1c). Resting target T cells were then challenged with cell-free HIV-1 (Fig. 1a). VS-priming significantly enhanced the number of Gag^+^ resting T cells, as measured by intracellular Gag staining (Fig. 1d and Extended Data Figs. 1d,e and 2a). Importantly, a substantial proportion of Gag^+^ resting T cells downregulated cell-surface CD4 (Gag^+^CD4^low^), confirming productive infection and viral gene expression rather than simply virus capture (Fig. 1d). Concordantly, inhibiting reverse transcription (RT) with efavirenz (EFV) completely abolished Gag positivity and CD4 downregulation (Fig. 1d). Hereafter, we refer to Gag^+^CD4^low^ cells as infected. Resting memory T cells, rather than naive cells, were preferentially infected (Extended Data Fig. 1f), consistent with previous reports and their selective infection in vivo^8,16,18,19^. We confirmed that enhanced infection of resting target T cells after VS-priming was not confounded by donor cells becoming infected with challenge virus by demonstrating that (1) donor T cell infection levels remained the same over the time-course of our experiments; (2) enhanced permissivity was evident when target cells were sorted after VS-priming to remove donor cells before virus challenge (Extended Data Fig. 1g–i); and (3) enhanced infection in VS-primed cells occurred using single-cycle pseudotyped ΔEnv virus (incapable of VS formation or spreading infection) as the challenge virus (Extended Data Fig. 1j,k). Enhanced infection of resting T cells was not due to classical T cell receptor (TCR) activation, as evidenced by failure to increase activation marker expression after VS-priming (Fig. 1e and Extended Data Fig. 1l–q) and the lack of any difference between VS-primed and non-primed cells in the percentage of Gag^+^ cells expressing CD69, CD25, HLA-DR or CD38 or the MFI (Fig. 1e and Extended Data Fig. 1l–o). Depleting CD25^+^ and CD69^+^ cells from the target cell population or infecting without IL-2 did not alter VS-priming (Extended Data Fig. 1r,s). VS-priming also enhanced infection of resting T cells challenged with the CCR5-tropic transmitter–founder viruses CH040 and CH077 (Fig. 1f) and both CXCR4- and CCR5-tropic GFP-reporter viruses (Fig. 1g and Extended Data Fig. 1t and Extended Data Fig. 2b,c).

**Extended Data Fig. 3:**
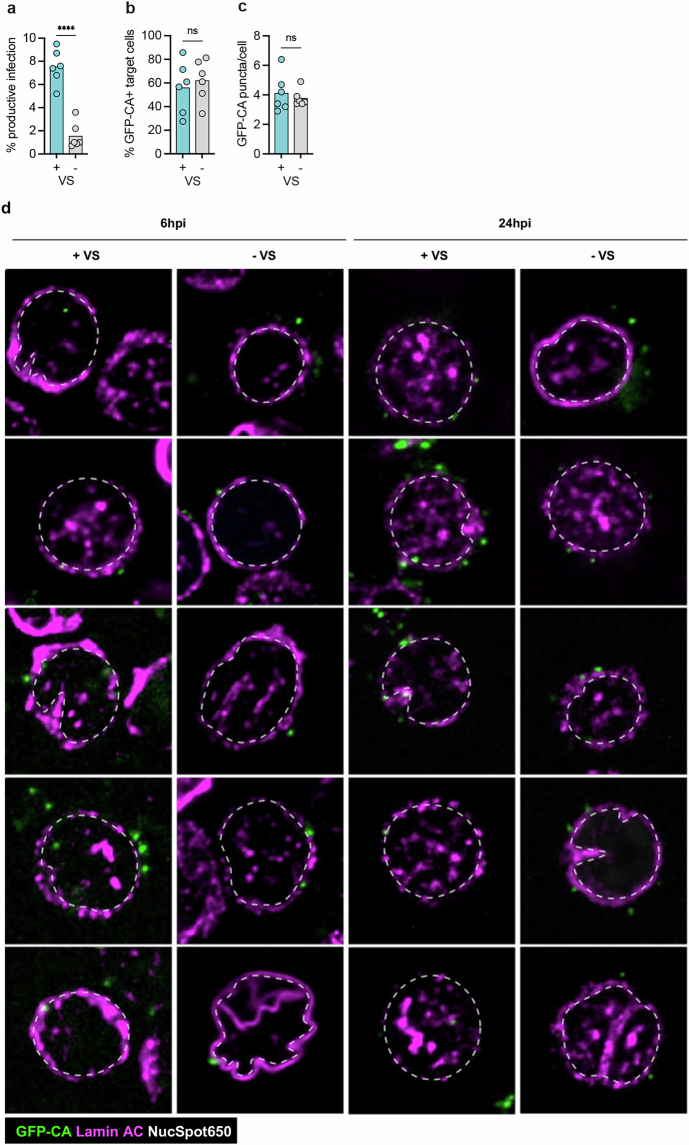
HIV-1 GFP-CA SR imaging, related to Fig. 1. (**a**) Productive infection of target T-cells with GFP-CA NL4.3 virus 72hpi, n = 6. Related to Fig. 1h–k. (**b**) Percentage of GFP-CA+ target T-cells 24hpi, n = 6. Related to Fig. 1i. (**c**) Number of GFP-CA puncta per GFP+ target T-cells 24hpi, n = 6. Related to Fig. 1j. (**d**) Super-resolution imaging of resting target T-cells +VS and −VS after challenge with HIV-1 GFP-CA virus (green) and co-stained for lamin A/C (magenta) at 6 and 24hpi. Dashed line outlines the nucleus based on NucSpot650 dye. Related to Fig. 1h–k. Mean and individual values from independent primary cell donors are shown. a-c) two-tailed paired t-test. ns, non-significant; *, *p* < 0.05; ****, *p* < 0.0001. Source data

**Extended Data Fig. 4:**
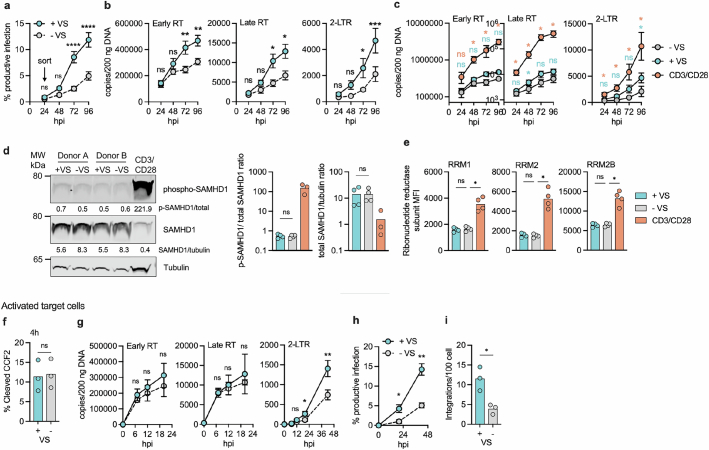
Related to Fig. 1. (**a-c**) Resting targets were recovered by flow sorting from co-cultures at 24hpi and quantified over time (24-96 hpi) for (**a**) productive infection and (**b**) qPCR for early and late RT products and 2-LTR circles, n = 3. (**c**) For comparison, cell-free infected CD3/CD28-activated cells were quantified and plotted alongside flow-sorted target cells (same data as in b). (**d**) Immunoblotting for phosphorylated and total SAMHD1 in resting targets 48hpi or after 72 h CD3/CD28 stimulation, n = 4. Related to Fig. 1r. (**e**) Expression of ribonucleotide reductase subunits RRM1, RRM2 and RRM2B in resting targets at 48hpi and after 72 h CD3/CD28 stimulation, n = 4.(**f-i**) Infected Jurkat donor cells were co-cultured with CD3/CD28 activated primary target T-cells for 24 h, n = 3. (**f**) Viral fusion 4hpi (cleaved CCF2). **(g-i**) Target cells were recovered by flow sorting from co-cultures and infected with HIV-1 NL4.3 and quantified for (**g**) early and late RT products and 2-LTR circles, (**h**) productive infection and (**i**) integration as in Fig. 1m–o. Mean ± SEM or individual values from independent primary cell donors are shown. a-c, g-h) two-way ANOVA with Šídák post-test; d, f, i) two-tailed paired t-test; e) one-way ANOVA with Dunnett post-test. ns, non-significant; *, *p* < 0.05; **, *p* < 0.01, ***, *p* < 0.001; ****, *p* < 0.0001. Source data

To directly visualize resting T cell infection, we performed instant structured illumination microscopy (iSIM) imaging of virions containing GFP-labelled capsid protein (GFP–CA)^20^ and confirmed infection enhancement by VS-priming (Extended Data Fig. 3a). VS-primed T cells were challenged with GFP–CA HIV-1 using the same MOI as in Fig. 1d–g and were imaged at 6 h and 24 h post-infection (h.p.i.; Fig. 1h–k and Extended Data Fig. 3). We detected on average two GFP–CA^+^ puncta per T cell, confirming low-multiplicity infection (Fig. 1j). VS-priming did not enhance virus fusion measured by GFP–CA^+^ puncta per cell (Fig. 1i–k and Extended Data Fig. 3b,c) or Blam-Vpr assay (Fig. 1l and Extended Data Fig. 2d). However, single-cell analysis of GFP–CA at 6 h.p.i. revealed a marked difference in subcellular localization, with VS-primed T cells showing significantly more capsids at the NE co-localizing with lamin A/C (31% primed versus 12% non-primed) and inside the nucleus (12% primed versus 2% non-primed) (Fig. 1k). In the non-primed condition, GFP–CA was almost exclusively outside the lamin A/C signal (86%). The nucleus-associated capsid signal in non-primed cells did not catch up over time and remained significantly lower at 24 h.p.i. (Fig. 1k and Extended Data Fig. 3), consistent with VS-primed cells maintaining their infection advantage (Fig. 1m). We next recovered dye-labelled resting target T cells by flow sorting and quantified infection over time (Fig. 1m and Extended Data Fig. 2e) and post-entry events (Fig. 1n,o). Concordant with enhanced NI, we observed a significant increase in 2-LTR circles (a surrogate marker for NI) at 48 h.p.i. in the VS-primed condition (Fig. 1n) and increased proviral integration (Fig. 1o). The percentage of infected cells increasing over time evidences sustained spreading infection (Fig. 1m). Infected resting T cells released virus into the culture supernatant (Fig. 1p) and spread infection to fresh resting T cells (Fig. 1q). The small but significant increase in early and late RT products at 48 h.p.i., which temporally coincides with 2-LTR circle measurements (Fig. 1n), is consistent with nuclear RT^20–23^. Again, RT and 2-LTR circles in non-VS primed targets did not catch up over time (Extended Data Fig. 4a,b), and activated T cells maintained their kinetic advantage (Extended Data Fig. 4c). Consistent with resting T cells not becoming activated, cellular dNTP levels remained the same between VS-primed and non-primed cells but were significantly increased in T cells activated with CD3 and CD28, as expected (Fig. 1r). VS-priming did not alter cellular dNTP regulators, measured by SAMHD1 phosphorylation and ribonuclease reductase (RRM1, RRM2, RRM2B) levels (Extended Data Fig. 4d,e). Adding exogenous deoxynucleosides to resting T cells boosted VS-primed and non-primed infection equally (twofold) and did not rescue non-primed infection to that of VS-priming (Fig. 1s). Importantly, infection of activated T cells, which have high levels of dNTPs (Fig. 1r), was also significantly enhanced by VS-priming and correlated with increased 2-LTR circles and integration, but not increased RT products (Extended Data Fig. 4f–i). These data suggest that RT is not the dominant rate-limiting step in T cell infection but, rather, VS-priming improves T cell permissivity by enhancing HIV-1 capsid NI, which poses a barrier to resting T cell infection.

We next investigated capsid NI in a more realistic model of CCS. To do this, activated primary T cells were infected with replication-competent HIV-1 and co-cultured with autologous resting target T cells to allow for CCS. In parallel, resting target T cells were challenged with high-MOI cell-free HIV-1 (in the absence of infected donors) to allow for a fair comparison of productive infection at equivalent virus dose (Fig. 1t). As expected, resting T cells were significantly more permissive to productive infection through CCS (Fig. 1u). iSIM imaging showed that a higher proportion of cell-associated capsids were localized inside the nucleus in the CCS condition compared with the cell-free condition (Fig. 1v–x). Significantly higher levels of 2-LTR circles but not RT products were detected in CCS compared with in cell-free infection (Fig. 1y). Thus, increased infection mediated by CCS is also associated with enhanced NI under conditions that more closely mimic in vivo infection dynamics.

## CD4 signalling enhances capsid import

**Fig. 2:**
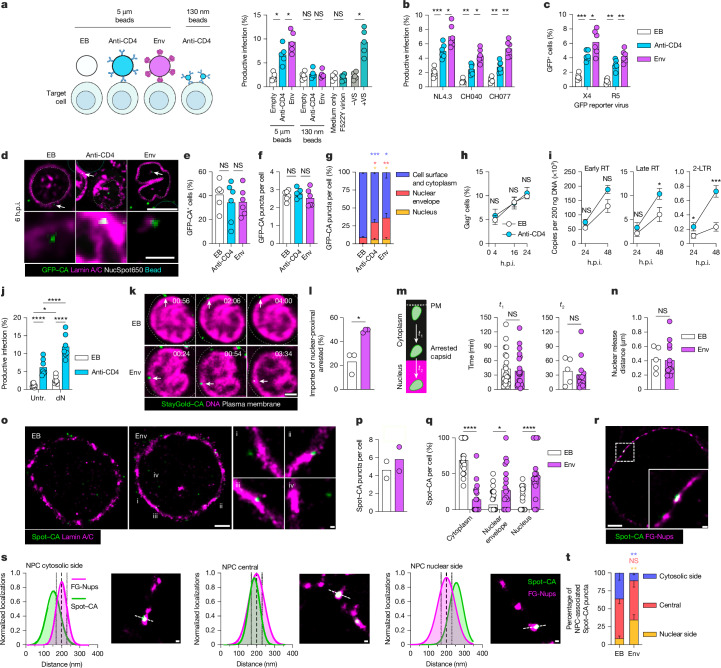
CD4 engagement mediates capsid NI enhancement. **a**, T cells were stimulated with beads (5 μm or 130 nm) coated with anti-CD4 antibodies, Env or left empty (EB), with Env-F522Y virions (1 h) or with or without VS-priming for 24 h before 72 h infection. *n* = 5. **b**,**c**, Productive infection with the indicated isolates (**b**) or GFP-reporter viruses (**c**). *n* = 6. **d**–**g**, Bead-stimulated cells were infected with GFP–CA virus. *n* = 6. **d**, Representative iSIM images. Scale bars, 5 µm (top) and 1 µm (bottom). **e**, GFP–CA^+^ cells. **f**, The average GFP–CA puncta per GFP^+^ cell. **g**, The GFP–CA distribution as in Fig. 1. Statistical comparisons are colour coded. **h**,**i**, Virus entry (Gag^+^) (**h**; *n* = 3) and early/late RT products and 2-LTR circles in targets (**i**; *n* = 3). **j**, Infection of dN-pretreated (24 h) or untreated cells. *n* = 7. **k**–**n**, mStayGold–CA virus live-cell imaging (time (min:s) after virus–cell association). Scale bar, 200 nm. *n* = 3. **l**, The percentage of imported capsids of total nuclear-proximal arrested capsids. *n* = 3 donors. **m**, The time from capsid entry at the plasma membrane (PM) to nuclear-proximal arrest (*t*_1_, *n* = 21 virions) and from arrest to NI (*t*_2_, *n* = 5 (EB) and *n* = 13 (Env) virions). **n**, The imported capsid distance from nuclear rim (µm). *n* = 5 (EB) and *n* = 13 (Env) virions. **o**–**t**, dSTORM imaging of Spot–CA (6 h.p.i.). **o**, Representative images of Spot–CA and lamin A/C. Scale bars, 1 µm (lower magnification) and 100 nm (higher magnification). **p**, Average Spot–CA puncta per cell. *n* = 2. **q**, Spot–CA localization; *n* = 22 (EB) and *n *= 17 (Env) cells from **o**. **r**, Representative image of Spot–CA and FG-Nups, from *n* = 3. Scale bars, 1 µm (lower magnification) and 100 nm (higher magnification). **s**, Spot–CA and FG-Nups normalized localization frequency along a single plane (white dashed line in images; representative histograms) at the cytosolic, central and nuclear side of the NPC. *n* = 38 (EB) and* n* = 48 (Env) virions. Scale bars, 100 nm. **t**, Quantification of NPC-associated Spot–CA puncta from **s**. Data are mean ± s.e.m. (**g**–**i**) and individual datapoints (**a**–**f** and **j**–**q**). Statistical analysis was performed using one-way ANOVA with Dunnett’s post test (**a**, **e** and **f**), two-way ANOVA with Šídák’s post test (**b**, **c**, **g**–**j**, **q** and **t**), paired two-tailed *t*-tests (**l** and **p**) and two-tailed Mann–Whitney *U*-tests (**m** and **n**). Unless indicated, 5 μm beads were used. The diagram in **m** was created using BioRender; Mesner, D. https://BioRender.com/ghqita5 (2026). Source data

**Extended Data Fig. 5:**
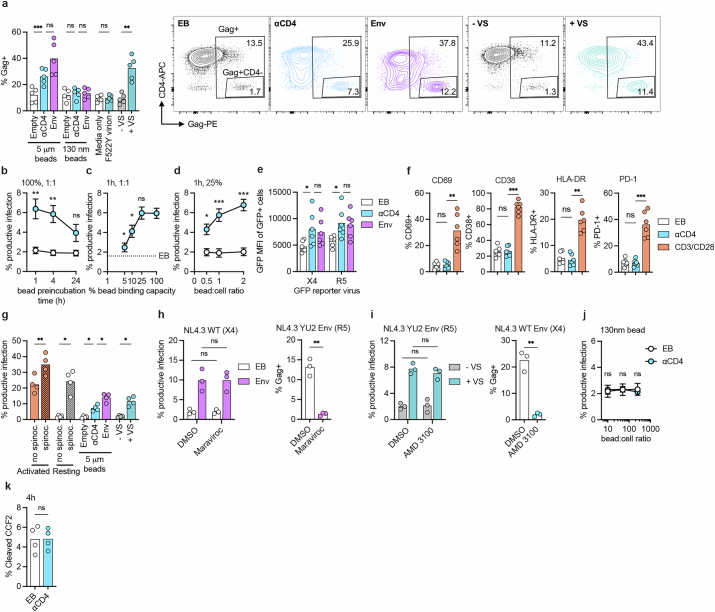
Related to Fig. 2. (**a**) Percent Gag+ cells incubated with beads, virions or donor cells as indicated and described in Fig. 2a, n = 5. Shown are representative dot plots of CD4 and Gag expression. (**b**) Productive infection of cells incubated for 1-24 h with empty (EB) or αCD4 5 µm bead at a 1:1 bead to cell ratio, n = 4. Beads conjugated at 100% binding capacity. (**c**) Productive infection of resting cells incubated with 5 µm beads for 1 h (1:1 ratio), n = 4. Beads were conjugated with increasing amounts of αCD4 Ab (5-100% binding capacity). (**d**) Productive infection of cells incubated with increasing doses of 5 µm beads for 1 h, n = 4. Beads conjugated at 25% binding capacity. (**e**) Expression of GFP (MFI) in GFP+ population of cells from Fig. 2c, n = 6. (**f**) Expression of CD69, CD38, HLA-DR or PD-1 in cells after 72 h bead incubation or CD3/CD28 stimulation, n = 6. (**g**) Productive cell-free infection (+/− spinoculation) of activated and resting cells compared to infection of bead- or VS-primed cells at 72 hpi, n = 4. (**h**) Productive infection of cells treated with DMSO or CCR5-antagonist Maraviroc and incubated with Env beads prior to infection with WT (X4-tropic) NL4.3 (left panel) or YU2 (R5-tropic) NL4.3 (right panel), n = 3. (**i**) Productive infection of cells treated with DMSO or CXCR4-antagonist AMD 3100 and VS-primed prior to infection with or YU2 (R5-tropic) NL4.3 (left panel) or WT (X4-tropic) NL4.3 (right panel), n = 3. (**j**) Productive infection of cells incubated with increasing doses of 130 nm beads for (conjugated at 100% binding capacity), n = 4. (**k**) Viral fusion at 4hpi measured as cleaved CCF2 substrate, n = 4. Mean ± SEM or individual values from independent primary cell donors are shown. a, c, f-g) one-way ANOVA with Dunnett post-test. b, d-e, h-i) two-way ANOVA with Šídák post-test. h, i, k) two-tailed paired t-test. ns, non-significant; *, *p* < 0.05; **, *p* < 0.01, ***, *p* < 0.001; ****, *p* < 0.0001. EB, empty bead; hpi, hours post-infection. Source data

CD4 complexes with the kinase LCK and signals in response to HIV-1 Env binding^24^, but whether this directly influences T cell permissivity to infection is unclear. We hypothesized that binding of CD4 on target T cells by Env expressed on infected donor T cells during cell–cell contact drives enhanced capsid NI and infection. To test this, 5 µm beads (a size similar to a T cell) were conjugated with either anti-CD4 antibody or HIV-1 Env-SOSIP trimer (CCR5 tropic) (Fig. 2a) and incubated with resting primary T cells (1:1 ratio) to mimic VS-priming before viral challenge. Cross-linking CD4 with either bead-bound CD4 antibody or Env significantly enhanced infection of T cells, achieving levels equivalent to cell–cell contact and VS-priming (Fig. 2a and Extended Data Fig. 5a,b). Increasing the number of beads incubated with T cells or increasing the amount of CD4 antibody conjugated to beads boosted permissivity in a dose-dependent manner (Extended Data Fig. 5c,d). Similar to VS-priming, bead stimulation enhanced infection by the transmitter–founder viruses CH040 and CH077 and by CXCR4- and CCR5-tropic GFP reporter virus (Fig. 2b,c and Extended Data Fig. 5e). Importantly, CD4 cross-linking did not activate resting T cells (Extended Data Fig. 5f). For comparison, we show the relative efficiency of different commonly used infection models side by side with VS and bead priming; as expected, activated T cells are most efficiently infected (Extended Data Fig. 5g). CXCR4 and CCR5 can themselves signal; however, T cells that were pretreated with maraviroc or AMD3100 (blocking CCR5-tropic or CXCR4-tropic Env binding, respectively) still displayed enhanced permissivity (Extended Data Fig. 5h,i), indicating that CD4 binding alone is sufficient to licence resting T cell infection. Importantly, neither cell-free virus nor 130 nm beads conjugated with anti-CD4 antibody or Env (which are a similar size to HIV-1 virions) enhanced infection (Fig. 2a and Extended Data Fig. 5j). In exact agreement with VS-priming (Fig. 1h–l), stimulating T cells with anti-CD4 or Env beads did not enhance HIV-1 fusion and entry (Fig. 2d–f,h and Extended Data Fig. 5k) but significantly increased HIV-1 GFP–CA at the NE or inside the nucleus (Fig. 2d–g). In fact, 37% of cells incubated with Env beads showed this phenotype compared with 9% of cells incubated with empty beads (Fig. 2g). Furthermore, anti-CD4-antibody-coated beads significantly increased 2-LTR circles and, to a lesser extent, RT products (Fig. 2i). Again, the addition of exogenous deoxynucleosides boosted infection, but anti-CD4-stimulated cells retained their infection advantage (Fig. 2j).

Next, we performed live-cell iSIM imaging to examine the overall kinetics of capsid NI in resting T cells (Fig. 2k–n). We defined successful NI as capsids colocalizing with the nuclear content DNA marker dye as previously done for live-cell imaging of HIV-1 in T cells^21^. We identified 22 cytoplasmic capsids that moved from the plasma membrane and arrested proximal to the nucleus after cell entry from imaging 150 cells incubated with empty beads, compared with 27 capsids from imaging 75 cells in the Env-bead condition. We imaged these capsids every 2 min over 4 h and measured the time for the arrested capsids to move into the nucleus. Significantly more capsids entered the nucleoplasm in Env-bead-primed cells compared with the empty-bead controls (49% versus 23%) (Fig. 2l and Supplementary Videos 1 and 2). However, the overall kinetics of import was similar (Fig. 2m). Once in the nucleoplasm, capsid localization appeared similar (Fig. 2n). This suggests that the block to NI in resting T cells is binary rather than kinetic, with CD4 signalling increasing the probability that a capsid will successfully import rather than significantly accelerating the process.

To analyse capsid interactions with the NE in resting T cells at super-resolution, we used dSTORM imaging. Resting T cells stimulated with Env beads or empty beads were challenged with HIV-1 expressing Spot-tagged CA, fixed and stained with lamin A/C to visualize the nuclear boundary (Fig. 2o). Similar numbers of capsids  in cells primed with Env beads and empty beads confirmed the equivalent virus dose (Fig. 2p). Quantification of capsid localization revealed that significantly more capsids were associated with the NE or inside the nucleus (imported) in cells stimulated with Env beads (Fig. 2q). To determine the position of capsids relative to the NPC, we performed two-colour dSTORM, imaging CA and phenylalanine-glycine-rich nucleoporins (FG-Nups) to resolve capsid–NPC interactions (Fig. 2r–t). We analysed capsids and their positions relative to the peak frequency of the FG-Nups localizations of the NPC^25^ and observed capsids in three positions at the NPC as previously reported^25^: cytosolic side, central and nuclear side. Quantification revealed that, while most capsids were localized in the central position of the NPC in both conditions, directly overlapping with the maximum FG-Nups signal, more were associated with the nucleocytoplasmic side of the NPC in Env-bead-stimulated cells compared with the empty-bead controls (35% versus 9%, respectively) suggesting enhanced translocation. By contrast, in the empty-bead controls, comparatively more capsids were on the cytoplasmic side (36% empty beads versus 11% Env beads). Our observation of numerous capsids associated with the nuclear side of the NPC is concordant with other studies^25,26^ and the notion that capsid release into the nucleoplasm is not instantaneous.

## CD4 signalling activates CDK1

**Fig. 3:**
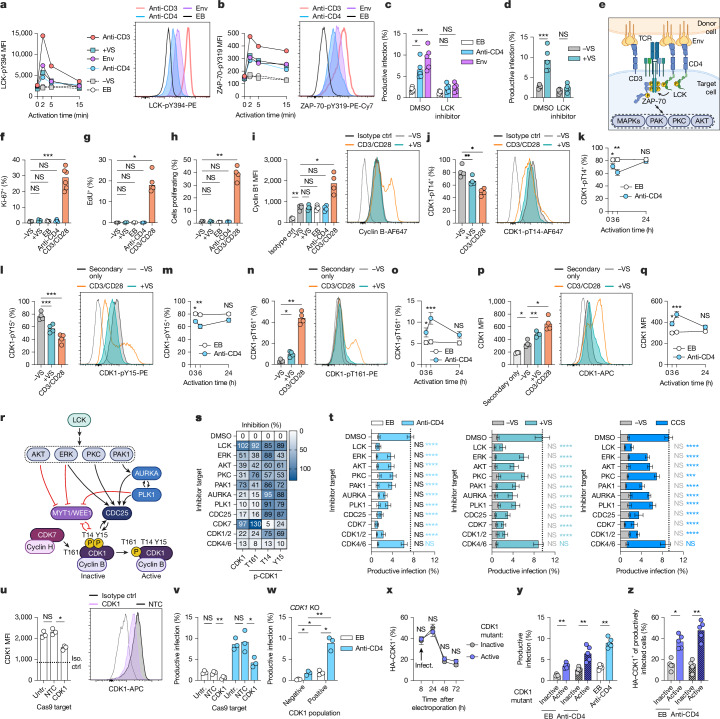
CDK1 activation by CD4–LCK signalling drives increased permissivity to infection. **a**,**b**, LCK phosphorylated at Tyr394 (LCK-pY394) (**a**) and ZAP-70-pY319 (**b**) in stimulated target cells. Representative graphs and histograms are shown from *n* = 4. **c**,**d**, Infection of bead-primed (**c**) or VS-primed (**d**) cells is LCK dependent. *n* = 5. **e**, Schematic of CD4 signalling activation. **f**–**q**, Pathway activation in targets with or without VS-priming (24 h), with bead treatment (up to 24 h) or with anti-CD3/CD28 stimulation (72 h). **f**–**h**, Ki-67 expression (**f**; *n* = 6), EdU-incorporation (**g**) and cell proliferation (72 h) (**h**; *n* = 4). **i**, Cyclin B1 expression in bead-stimulated (24 h) or CD3/CD28-stimulated (72 h) targets. *n* = 4.** j**–**q**, Phosphorylation of CDK1 at Thr14 (**j**,**k**), Tyr15 (**l**,**m**) and Thr161 (**n**,**o**) or total protein levels of CDK1 (**p**,**q**). Representative histograms are shown. *n* = 4. **r**, Signalling pathways activating CDK1. **s**, Anti-CD4-bead-stimulated (6 h) T cells were pretreated with the indicated inhibitors. The heat map shows the percentage inhibition normalized to the DMSO control. *n* = 4. **t**, Productive infection of targets stimulated with beads or donor cells (with or without VS or CCS) and pretreated with inhibitors. *n* = 4. Bars are superimposed. **u**–**w**, Resting T cells were electroporated with CDK1 or non-targeting control (NTC) Cas9–RNA complexes. *n* = 3. **u**, CDK1 (MFI) expression after CRISPR knockout (KO). **v**, Productive infection of bead-stimulated *CDK1-*knockout cells (72 h.p.i.) (15 days after electroporation). **w**, Infection levels in the CDK1-positive or CDK1-negative population in *CDK1*-knockout cells from **v**. **x**–**z**, Resting T cells were electroporated with mRNA encoding active (T14A/Y15F) or inactive (T14A/Y15F/T161A) HA–CDK1 mutant. **x**, The percentage of cells expressing HA–CDK1 mutants. *n* = 3. **y**, Productive infection of bead-stimulated mRNA-electroporated cells at 72 h.p.i. *n* = 5. **z**, HA–CDK1 expression in productively infected cells from **y**. Data are mean ± s.e.m. or individual values from independent primary cell donors. Isotype control (control) or secondary antibody only show background staining. Statistical analysis was performed using one-way ANOVA with Dunnett’s post test (**f**–**j**, **l**, **n**, **p**, **u**, **y** and** z**) and two-way ANOVA with Šídák’s post test (**c**, **d**, **k**, **m**, **o**, **q**, **t** and **v**–**x**). The diagrams in **e** and** r** were created using BioRender; Mesner, D. https://BioRender.com/ghqita5 (2026). ctrl, control; MFI, mean fluorescence intensity. Source data

**Extended Data Fig. 6:**
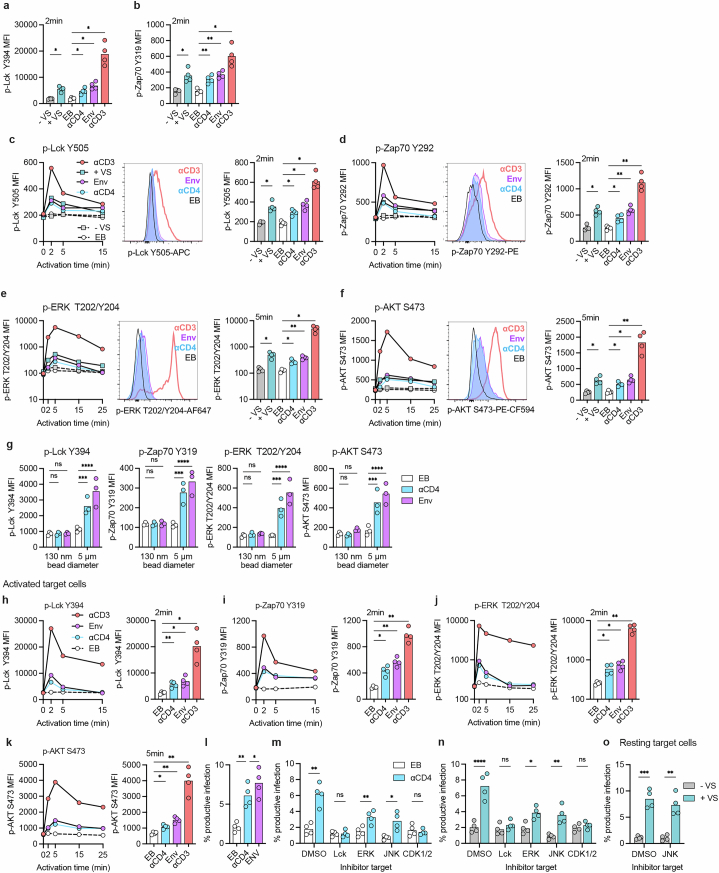
Lck-dependent CD4-signalling is triggered in resting an activated target T-cells, related to Fig. 2. (**a-b**) Resting target cells were incubated with beads or donor cells (+VS, −VS) and phosphorylation of (**a**) Lck Y394 and (**b**) Zap70 Y319 was measured by phosphoflow at 2 min, n = 4. Related to Fig. 3a–b. **(c-f**) Resting target cells were incubated with indicated beads or donor cells (+/−VS) and phosphorylation of (**c**) Lck Y505, (**d**) Zap70 Y292, (**e**) ERK T202/Y204 and (**f**) AKT S473 was measured by phosphoflow at 0-25 min. Left and middle panels show representative curves and histograms. Right panels show quantification at 2 min or 5 min activation time as indicated, n = 4. **(g)** Phosphorylation of Lck Y394 and Zap70 Y319 at 2 min and ERK T202/Y204 and AKT S473 at 5 min after stimulation with 130 nm or 5 um beads at 1:50 and 1:1 cell:bead ratio, respectively, n = 3. (**h-k**) CD3/CD28 activated target cells were incubated with beads (5 µm beads at 25% binding and 1:1 bead:cell ratio) and phosphorylation of (**h**) Lck Y394, (**i**) Zap70 Y319, (**j**) ERK T202/Y204 and (**k**) AKT S473 was measured by flow cytometry at 0-25 min. Left panels show representative curves and right panels show quantification at 2 min or 5 min activation time as indicated, n = 4. (**l**) Productive infection of activated target cells from (h-k), n = 4. (**m**,**n**) Productive infection of activated target cells incubated with inhibitors and (**m**) beads or (**n**) autologous infected donor cells (+/−VS), n = 4. (**o**) Productive infection of resting target cells incubated with JNK inhibitor and autologous infected donor cells (+/−VS), n = 4. Activated target cells were infected for 24 h and resting target cells for 72 h; both with WT NL4.3. Mean and individual values from independent primary cell donors are shown. a-f, h-k) one-way ANOVA with Šídák post-test. l) one-way ANOVA with Dunnett post-test. **g**, m-o) two-way ANOVA with Šídák post-test. ns, non-significant; *, *p* < 0.05; **, *p* < 0.01, ***, *p* < 0.001; ****, *p* < 0.0001. EB, empty bead; MFI, mean fluorescence intensity. Source data

Enhanced capsid NI and infection in response to CD4 engagement suggests signalling activation. To test this idea, resting T cells incubated with either anti-CD4 or Env 5-µm beads or stimulated through VS-priming were analysed by phospho flow cytometry. Indicative of CD4 signalling activation, we observed a rapid and transient peak of LCK phosphorylation (Fig. 3a and Extended Data Fig. 6a,c) as well as ZAP-70 (the downstream target of LCK) (Fig. 3b and Extended Data Fig. 6b,d), ERK and AKT (Extended Data Fig. 6e,f). Smaller virus-sized 130 nm beads that failed to enhance infection did not activate CD4 signalling (Extended Data Fig. 6g). As expected, the magnitude of CD4-induced signalling was lower than TCR–CD3 cross-linking (Fig. 3a,b and Extended Data Fig. 6a–f) and did not activate T cells (Extended Data Fig. 5f). Importantly, inhibiting LCK abolished T cell infection enhancement induced by anti-CD4 or Env beads or VS-priming (Fig. 3c,d), functionally linking CD4 signalling to T cell permissivity (Fig. 3e). Anti-CD4 or Env beads also enhanced infection of activated T cells in a signalling-dependent manner (Extended Data Fig. 6h–o). CD4 engagement alone is therefore necessary and sufficient to enhance capsid NI, with level of CD4 engagement and signalling needed to make resting T cells permissive achieved through cell–cell contact at the VS, but not cell-free virus. Thus high-efficiency infection during CCS and T cell permissivity to HIV-1 are universally linked to activation of CD4 signalling by cell–cell contact, driving a transient permissivity enhancement.

**Extended Data Fig. 7:**
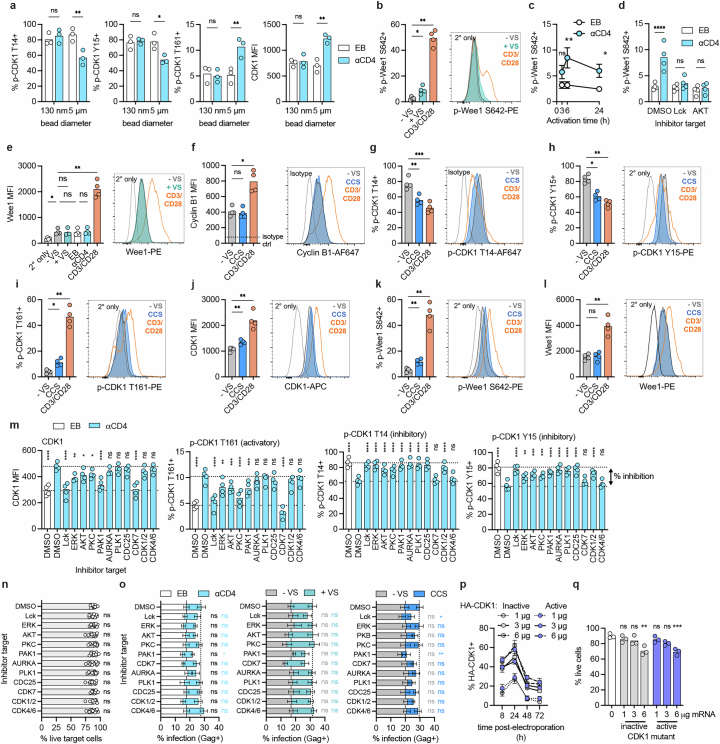
Related to Fig. 3. (**a**) Phosphorylation of CDK1 at residues T14, Y15 and T161 and total level of CDK1 in cells stimulated with indicated beads for 6 h, n = 3. (**b-d**) Phosphorylation of Wee1 S642 in target cells, n = 4. (**b**) Targets were incubated with **(b)** donor cells for 24 h (+/−VS) or CD3/CD28 stimulated for 72 h; **(c)** indicated beads for 3-24 h or (**d**) indicated inhibitors and beads for 6 h. (**e**) Expression of Wee1 in target cells incubated with beads or donor cells for 24 h or CD3/CD28 stimulated for 72 h, n = 4. (**f-l**) Target cells were incubated with donor cells (-VS or CCS) for 6 h or CD3/CD28 stimulated for 72 h and analysed by flow cytometry for (**f**) total cyclin B1, (**g**) phospho-CDK1 T14, (**h**) phospho-CDK1 Y15, (**i**) phospho-CDK1 T161, (**j**) total CDK1, (**k**) phospho-Wee1 S642, and (**l**) total Wee, n = 4. In cell-cell spread (CCS) condition, donor cells are infected with WT NL4.3 virus allowing for infection of target T-cells at the VS and no exogenous virus was added. (**m**) Target cells were pre-treated with inhibitors and incubated with αCD4 beads for 6 h to measure levels of total CDK1 and CDK1 phosphorylation at residues T14, Y15 and T161 as in Fig. 3, n = 4. Dashed lines indicate means of EB and αCD4 DMSO controls. Difference in MFI or percentage between αCD4 bead and EB DMSO controls represents 100% change used to calculate percent inhibition of CD4 bead activity by individual inhibitors as shown in Fig. 3s. (**n**) Percent live target cells after 72 h inhibitor treatment, n = 12. (**o**) Infection (Gag +) in target cells from Fig. 3t, n = 4. Bars are superimposed. (**p-q**) Resting cells were electroporated with increasing doses of HA-CDK1 mRNA and assed for (**p**) CDK1 expression and (**q**) cell viability at 24 h post-electroporation, n = 3. Related to Fig. 3x–z. Mean ± SEM or individual values from independent primary cell donors are shown. Isotype control or secondary antibody only show background staining. a, d-k, m, q) one-way ANOVA with Dunnett post-test. b-c, l, n) two-way ANOVA with Šídák post-test. ns, non-significant; *, *p* < 0.05; **, *p* < 0.01, ***, *p* < 0.001; ****, *p* < 0.0001. EB, empty bead; hpi, hours post-infection; MFI, mean fluorescence intensity. Source data

We hypothesized that CD4 signalling alters the NPC to enhance capsid NI by targeting CDK1—the main kinase that phosphorylates Nups to trigger NPC remodelling and disassembly before mitosis^12,27^. First, we confirmed that CD4 stimulation alone does not trigger classical cell-cycle entry and mitosis, measured by the lack of Ki-67 expression, DNA synthesis or cell proliferation (Fig. 3f–h). While CDK1 activity is generally associated with proliferating cells (through accumulation of cyclin B during the S and G2 phases of the cell cycle), we found that resting T cells do express low but detectable levels of cyclin B (Fig. 3i). CDK1 activity is also controlled by a complex regulatory network involving phosphorylation-dependent activation by CDK7, inhibition by WEE1 and MYT1, and activation by dephosphorylation through CDC25^28,29^. Notably, VS-priming or anti-CD4 5-µm beads activated CDK1, as evidenced by loss of inhibitory phosphorylation at Thr14 and Tyr15 and increased activating phosphorylation at Thr161 (Fig. 3j–o). By contrast, 130 nm beads did not activate CDK1 (Extended Data Fig. 7a). Importantly, CD4 engagement also increased inhibitory phosphorylation of WEE1 kinase at Ser642, preventing WEE1 from inhibiting CDK1 activity by phosphorylation of Tyr15 (Extended Data Fig. 7b–e). CD4 signalling also increased CDK1 levels in resting T cells (Fig. 3p,q). As expected, CD3–CD28 cross-linking that activates T cells and triggers mitosis more strongly modulated CDK1 phosphorylation and CDK1 and cyclin B protein levels (Fig. 3i,j–p). Importantly, the same changes in CDK1 were detected in resting target T cells during CCS at the VS (Extended Data Fig. 7f–l), linking CDK1 activation to high-efficiency infection by cell–cell transmission.

These data suggest a model of non-classical, cell-cycle-independent CDK1 activation through CD4–LCK signalling. Concordantly, pharmacologically blocking LCK signalling or downstream aurora A (AURKA), PLK1, CDC25 and CDK7 (Fig. 3r) inhibited CDK1 activation (Fig. 3s and Extended Data Fig. 7m). Inhibiting the upstream kinases ERK, AKT, PKC and PAK1 had smaller effects, probably reflecting pathway redundancy. Inhibiting CDK4/6, which regulates cell-cycle entry in G1 and is not linked to NPC remodelling, had no effect (Fig. 3s and Extended Data Fig. 7m). Directly linking the CD4–LCK–CDK1 axis to T cell permissivity, inhibiting LCK, CDC25, CDK7 and CDK1/2 all potently blocked productive infection enhancement in resting T cells (Fig. 3t). Targeting other kinases had intermediate effects and CDK4/6 inhibition had no effect (Fig. 3t and Extended Data Fig. 7n,o). Critically, depleting *CDK1* in resting T cells using CRISPR knockout significantly impaired HIV-1 infection (Fig. 3u–w), while electroporating resting T cells with mRNA encoding an active CDK1 mutant (T14A/T15F/T161) increased infection compared with cells expressing a CDK1 inactive mutant (T14A/T15F/T161A) (Fig. 3x–z and Extended Data Fig. 7p,q). Notably, in knockout and overexpression experiments, HIV-1 infection was predominantly within the CDK1^+^ population (Fig. 3w) or in cells expressing active CDK1 (Fig. 3q). Collectively, our results support a mechanism for CD4–LCK signalling driving CDK1 activation to enhance HIV-1 infection without triggering cell-cycle progression and cell division.

## CDK1 remodels NPCs for capsid import

**Fig. 4:**
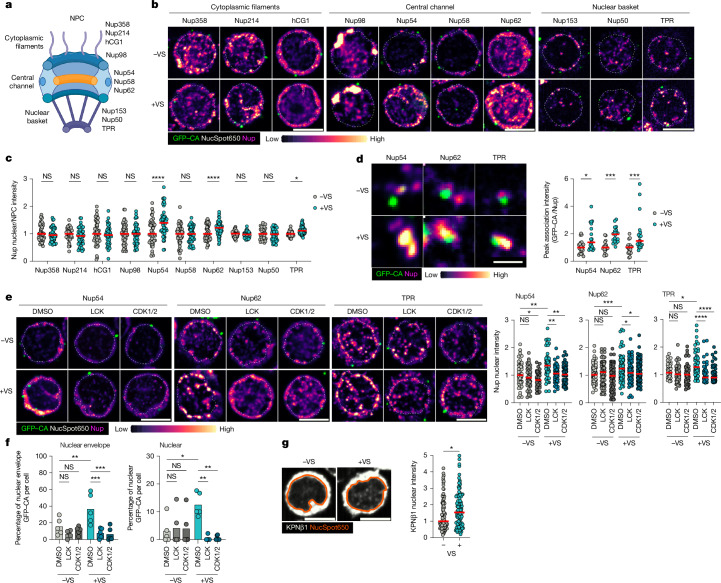
CD4–LCK signalling triggers nuclear pore remodelling and enhances nuclear transport. **a**, Schematic showing NPC organization with Nup358, Nup214, hCG1 (cytoplasmic filaments), Nup98, Nup62, Nup58, Nup54 (central channel), Nup153, Nup50 and TPR (nuclear basket). **b**, Representative images of Nup localization (magenta) and GFP–CA (green) in target T cells at 6 h.p.i. from *n* = 3. The dashed line indicates the nuclear border based on NucSpot650 dye. The intensity of Nup staining is pseudocoloured from lowest to highest (black–magenta–yellow gradient). Scale bars, 5 µm. **c**, Quantification of Nup nuclear/NPC intensity. *n* = 50 cells from 3 donors. **d**, Higher-magnification images showing the localization of GFP–CA with Nup54, Nup62 or TPR puncta in cells from **b**. Scale bar, 1 µm. Quantification shows peak Nup54, Nup62 or TPR pixel intensity in GFP–CA-associated puncta. *n* = 20 puncta from 3 donors. **e**, Representative images of Nup54, Nup62 and TPR localization in target T cells after LCK or CDK1/2 inhibitor treatment at 6 h.p.i. Quantification shows Nup nuclear intensity for each condition normalized to the median of DMSO without VS. *n* = 50 cells from 3 donors. Scale bars, 5 µm. **f**, NE or nuclear localization of GFP–CA in target T cells treated as in **e**. *n* = 5 donors. **g**, KPNβ1 nuclear intensity in target T cells incubated with donor cells for 24 h in the presence of exportin-1 inhibitor. *n* = 100 cells from 3 donors. Scale bars, 5 µm. The scatter dot plots in **c**–**e** and **g** show the median and individual measurements. The bar plots in **f** show mean and values from individual donors. Statistical analysis was performed using two-way ANOVA with Šídák’s post test (**c** and **d**), Kruskal–Walis tests with Dunn’s post test (**e**), one-way ANOVA with Šídák’s post test (**f**) and two-tailed Mann–Whitney *U*-tests (**g**). The diagram in **a** was created using BioRender; Mesner, D. https://BioRender.com/ghqita5 (2026). Source data

**Fig. 5:**
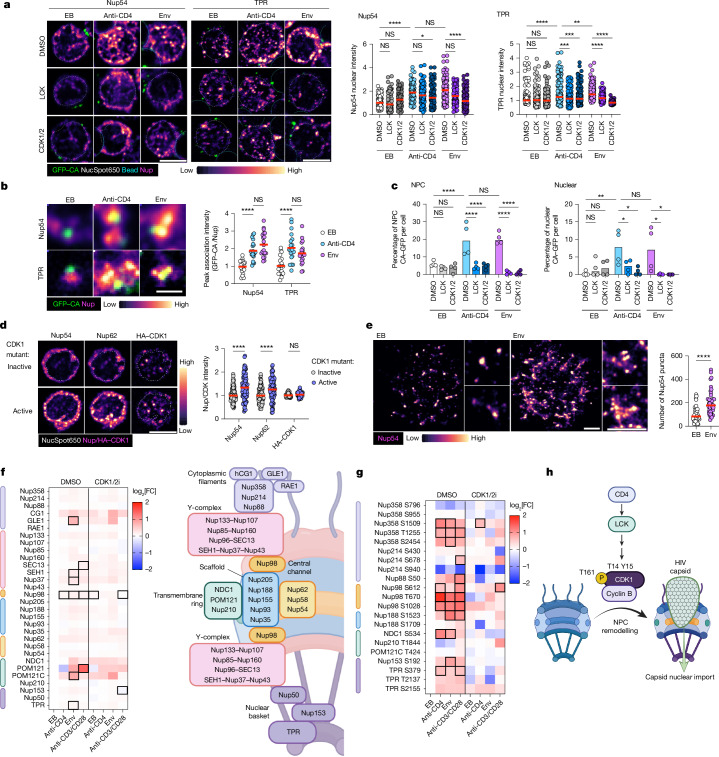
CD4 signalling activates CDK1 to remodel NPC architecture. **a**, Representative iSIM images of Nup54 and TPR in LCK- or CDK1/2-inhibited and bead-stimulated cells (6 h.p.i.). *n* = 100 cells from 3 donors. Scale bars, 5 µm. Quantification of Nup nuclear intensity normalized to the median of EB DMSO. **b**, Association of GFP–CA with Nup54 and TPR puncta in cells from **a**. Quantification of peak Nup54 or TPR pixel intensity in GFP–CA-associated puncta. *n* = 20 cells from 3 donors. Scale bar, 5 µm. **c**, NPC or nuclear localization of GFP–CA in cells treated as in **a**.* n* = 200 cells from 4 donors. **d**, Localization of Nup54, Nup62 and HA–CDK1 mutants at 6 h after electroporation in cells from Fig. 3x. Quantification of each Nup/CDK1 intensity is normalized to the median of the inactive mutant. *n* = 100 from 3 donors. Scale bar, 5 µm. **e**, dSTORM imaging of bottom-plane field of view Nup54 puncta in 6 h bead-stimulated cells. Localizations are pseudocoloured from lowest to highest (black–magenta–yellow gradient). Scale bars, 1 µm. Quantification of the number of Nup54 puncta per cell is shown. *n* = 46 cells from 6 donors. **f**,**g**, Total abundance (**f**) and phosphoproteomics analysis (**g**) of NPC components in 6 h bead-stimulated cells treated with CDK1/2 inhibitor. Quantification shows the log_2_-transformed fold change (FC) compared with the EB DMSO control. The bold cell border indicates significant changes (adjusted *P* < 0.05). *n* = 4 donors. Schematic of the NPC structure shows the components of each subcomplex. **h**, Model showing CD4–LCK signalling leading to CDK1 activation to remodel the NPC and increasing HIV-1 CA NI. The scatter dot plots in **a**,** b** and **d** show median and individual measurements. The bar plots in **c** show mean and values from individual donors. Statistical analysis was performed using Kruskal–Walis tests with Dunn’s post test (**a**), two-way ANOVA with Šídák’s post test (**b** and **d**), one-way ANOVA with Šídák’s post test (**c**) and two-tailed Mann–Whitney *U*-tests (**e**–**g**). The diagrams in **f** and **h** were created using BioRender; Mesner, D. https://BioRender.com/ghqita5 (2026). Source data

**Extended Data Fig. 8:**
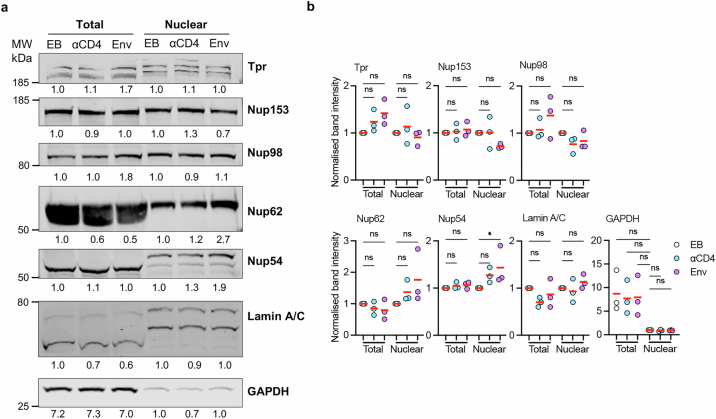
Nucleoporin levels in whole-cell and nuclear extracts, related to Fig. 5. (**a**) Representative immunoblots of Tpr, Nup153, Nup98, Nup62, Nup54, lamin A/C and GAPDH levels in total cell lysate and corresponding nuclear fraction from cells stimulated with beads for 6 h from n = 3. (**b**) Quantification shows band densitometry values normalized to EB control. Shown are mean and individual values, n = 3. Kruskal-Wallis test with Dunn’s post-test. ns, non-significant. Source data

To directly test whether the NPC responds to signalling triggered by cell–cell contact, we used iSIM to visualize Nups that localize to the three major NPC domains: cytoplasmic filament Nups (Nup358, Nup214, hCG1), FG-repeat enriched central channel Nups (Nup98, Nup54, Nup58, Nup62) and nuclear basket Nups (Nup153, Nup50, TPR) (Fig. 4a,b). This includes CA-binding Nups that act as co-factors for HIV-1 infection (Nup358, Nup98, Nup153, Nup62)^22,30–32^ as well as TPR and Nup62, which regulate NPC structure and assembly^33^. VS-priming significantly increased the intensity of Nup54, Nup62 and TPR punctate staining at the NE of resting T cells (Fig. 4b,c), indicative of dynamic changes to Nup localization and nuclear pores. HIV-1 GFP–CA was more strongly associated with Nup54-, Nup62- and TPR-rich puncta at the NE in VS-primed cells (Fig. 4d), suggesting that capsids may preferentially localize with these structures. Inhibiting LCK and CDK1/2 abrogated Nup54, Nup62 and TPR remodelling (Fig. 4e) and significantly reduced HIV-1 GFP–CA localization at the NE and NI in VS-primed cells (Fig. 4e,f). Cellular karyopherins interact with FG-repeat rich Nups to transport cargo through the central NPC channel and overcome the size-selective barrier to nucleocytoplasmic trafficking. Recent studies demonstrated that HIV-1 capsid acts as a karyopherin, recruiting FG-repeats in the NPC permeability barrier and obviating the need for an additional karyopherin to mediate NI^31,32^. We reasoned that, if VS priming was remodelling Nups and altering the NPC to enhance HIV-1 capsid NI, it should also increase the NI of cellular karyopherins. Concordantly, we observed a significant increase in the amount of karyopherin β1 (KPNβ1/importin β1) in the nucleus of resting T cells that had been VS-primed but not challenged with HIV-1 (Fig. 4g) (evident as white puncta inside the nuclear rim), consistent with cell–cell contact remodelling nuclear pores to enhance import of both viral and cellular cargo. Activation of CD4 signalling alone was sufficient to trigger formation of Nup54- and TPR-enriched nuclear puncta (Fig. 5a and Extended Data Fig. 8a,b) at which HIV GFP–CA co-localized (Fig. 5b). Inhibitor treatment showed that Nup changes and associated enhancement of capsid import were LCK and CDK1 dependent (Fig. 5a,c), linking activation of CD4–LCK signalling and CDK1 with dynamic changes to nuclear pores and enhanced infection. Evidencing a direct link between CDK1 and Nup remodelling, expressing an active CDK1 mutant (Fig. 3x–z) in resting T cells was sufficient to induce Nup54- and Nup62-enriched puncta, bypassing the need for CD4 signalling (Fig. 5d). Super-resolution dSTORM imaging showed a significant increase in the number of Nup54 puncta in resting T cells in response to CD4-induced priming (Fig. 5e).

**Extended Data Fig. 9:**
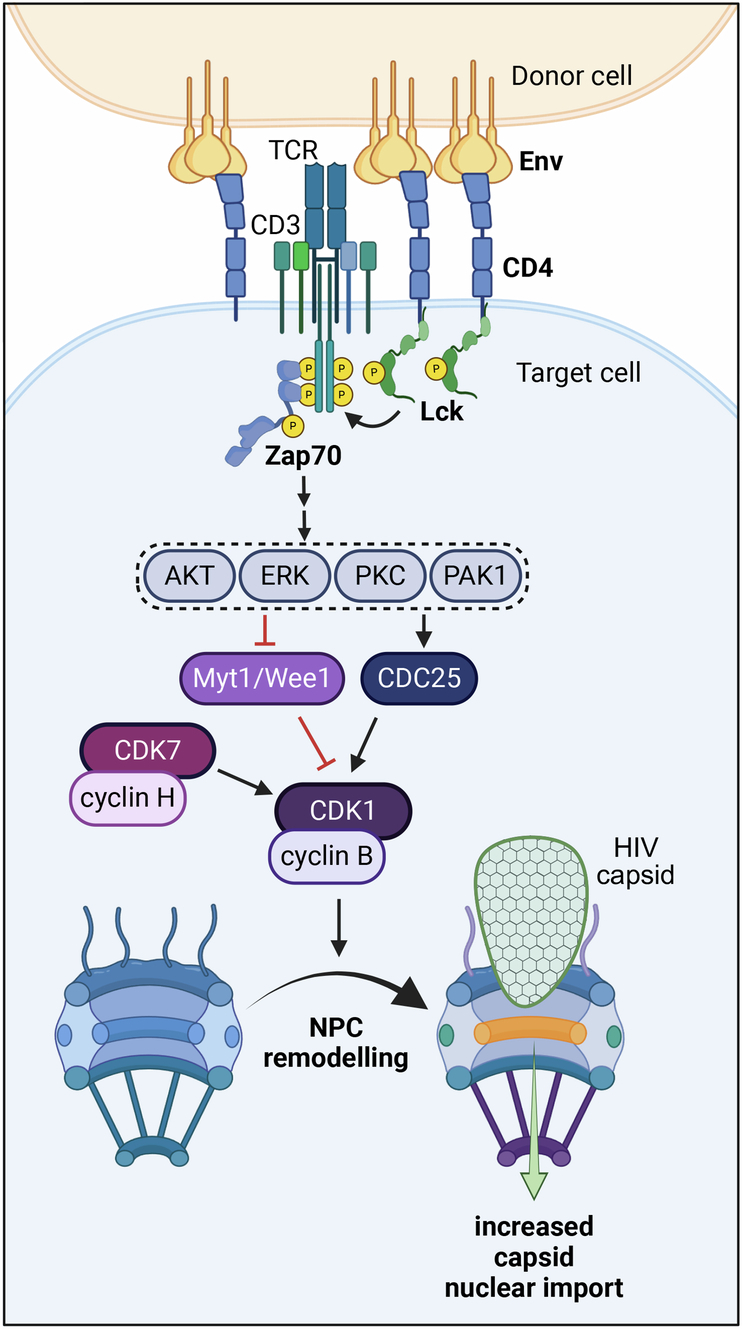
Summary model. Diagram depicts how cell-cell contact activates T-cell signalling to enhance HIV-1 capsid nuclear import and infection by nuclear pore remodelling. Created with BioRender.com.

To define the mechanism and test whether CDK1 was driving NPC remodelling through Nup phosphorylation, we performed mass spectrometry (MS) analysis to examine the Nup proteome and phosphoproteome in response to CD4 signalling. Resting T cells from four independent donors were stimulated for 6 h with empty bead control, anti-CD4 beads, Env beads, or anti-CD3 and anti-CD28 beads, in the presence or absence of CDK1/2 inhibitor, and analysed using quantitative MS. Bead priming and inhibitor treatment did not globally alter Nup abundance over 6 h (Fig. 5f, Supplementary Table 1 and Extended Data Fig. 8), suggesting that CD4-signalling-induced changes to Nup54, Nup62 and TPR intensity and puncta formation (Figs. 4b–f and 5a,b) are probably due to Nup relocalization, rather than increased protein expression. However widespread changes to Nup phosphorylation in response to CD4 signalling were observed in cytoplasmic, central channel and nuclear-basket Nups, with many sites showing increased phosphorylation (Fig. 5g and Supplementary Table 2). Signalling induced by CD4 and Env beads targeted the same Nups and phosphorylation sites. Treating cells with CDK1/2 inhibitor abrogated global Nup phosphorylation, abolishing essentially all significant Nup phosphorylation changes induced by CD4 signalling. These data suggest a mechanism of CDK1 driving NPC remodelling through Nup phosphorylation after CD4 signalling to licence HIV-1 capsid import by altering the NPC (Fig. 5h and Extended Data Fig. 9).

## Discussion

HIV-1 capsid NI delivers the viral genome for integration and is essential for infection. Here we show that resting T cells are highly refractory to HIV-1 because the step of capsid import is inefficient, presenting a barrier to successful infection. However, HIV-1 alleviates this block by using CCS to trigger CD4–LCK signalling and CDK1 activation that drives more efficient capsid NI to licence infection of resting T cells.

Activated T cells are highly susceptible to HIV-1 infection and T cell activation, and TCR engagement has therefore long been assumed to be necessary for infection. However, in vivo, most T cells are resting, not activated and have not previously been stimulated by antigen-specific TCR signalling. The source of HIV-1-infected resting T cells in vivo has been debated^1–3,8^ and largely attributed to previous infection of activated T cells that returned to a resting state^34^. Our finding of a role for CCS and CD4 signalling in enhancing infection through NPC remodelling offers an explanation for these observations. This is one way in which CCS drives increased infection and resting T cell permissivity. In vivo, the high density of immune cells in lymphoid tissue provides an ideal environment for CCS, where contact between infected and uninfected T cells^16,35,36^ can trigger synaptic CD4 signalling at the VS. By contrast, cell-free infection does not trigger sufficient CD4 signalling to activate CDK1 and licence efficient NI, explaining why resting T cells are largely refractory to infection. It is well established that CCS is significantly more efficient than cell-free infection^13–16^ and, by evidencing enhanced NI in both resting and activated T cells, we provide a paradigm to explain this. We conclude that CCS dominates not simply because more virus is transferred to the target T cell, but also because cell–cell contact triggers increased T cell permissivity overcoming rate-limiting steps to infection. Cytokine exposure can also trigger infection of resting T cells^37^ and future work to dissect the contribution of the immune microenvironment, including cytokines, to the molecular architecture of the NPC and capsid NI is warranted. Other medically important human viruses, including hepatitis B and herpesviruses, must also navigate the NPC barrier and are known to use CCS to most efficiently spread between cells^38^. Thus, evolution of viral CCS to activate signalling and target nuclear transport may well be a mechanism used by other pathogens to enhance infection and here we provide a framework to address this.

We evidence events at the cell surface directly affecting molecular events at the NE and regulation of the NPC by HIV-1. The NPC is a complex macromolecular machine comprising over 30 different Nups and 500 individual proteins, forming a 120 MDa size-selective channel in mammalian cells that regulates nucleocytoplasmic transport^11^. We propose a model in which HIV-1-induced CD4 signalling enhances NI because the NPC acts as a sensor that can respond to physiological cues and remodel to better accommodate large cargo^39^ like HIV-1 capsid, allowing the intact or largely intact capsid core to translocate through and release the reverse transcribed viral cDNA for integration. This model also offers a mechanistic explanation for how HIV-1-induced CD4 signalling influences cellular gene expression^40,41^, by also enhancing nucleocytoplasmic transport of cellular karyopherins. Both the structure of the NPC and some Nups themselves are known to be dynamic and rearrange in response to environmental stimuli^12,33,39^—in this case, HIV-1 Env binding to CD4 that targets the CDK1–Nup axis to remodel nuclear pores. Having observed wide-spread NPC remodelling associated with enhanced CA NI, we argue that the notion that only capsid-binding Nups are HIV-1 co-factors should be reconsidered. We instead propose that the entire NPC acts as a co-factor.

CDK1 is best known to regulate mitotic entry, but it also regulates many other diverse cellular functions through quantitative changes in CDK activity^29,42^. For example, CDK1 is the key kinase that drives the very early steps of NPC formation during interphase^43^ and the main kinase that phosphorylates Nups to trigger NPC remodelling^12,27^. Concordantly, we show that CD4 signalling and CDK1 activation drives widespread changes to Nup phosphorylation, including cytoplasmic, central-channel and nuclear-basket Nups, and alters Nup54, Nup62 and TPR localization in resting T cells. Although it remains unclear how NPC remodelling increases capsid import, changes to Nup phosphorylation and NPC architecture could enhance interactions between capsid and the NPC, and/or NPC permeability to cargo, improving capsid association with the NPC and subsequent translocation^31,32^, as we observed. The Nup58–Nup54–Nup62 complex contributes to forming the NPC-selective barrier and interacts with transport receptors for cargo import^11^. Nup62 and TPR are central regulators of NPC structure and assembly^33^ with Nup62 also binding to HIV-1 CA^31,44^. Whether Nup54/Nup62/TPR-enriched puncta associated with capsids represent nuclear pores that have reorganized to facilitate enhanced capsid passage into the nucleus, or nascent NPCs being assembled in response to external stimuli, remains an open question requiring finer nanoscale analysis of NPC architecture. Whether CD4 signalling affects capsid transport to the NPC by modulating the cytoskeletal network also warrants future investigation. Our results also raise interesting fundamental questions about nuclear pore architecture and regulation in primary human T cells — for example, the question of how nuclear pores compare at the ultrastructural level between resting and activated T cells and whether they change in response to other immune stimuli to influence T cell function. Given that Nups can directly regulate the spatial organization of chromatin proximal to nuclear pores^45,46^, we hypothesize that signalling-induced Nup/NPC remodelling may also impact HIV-1 integration site selection in T cells^47–49^ infected through CCS and cellular gene expression, with consequences for T cell function, HIV-1 persistence and pathogenesis.

Our findings have broader implications beyond infection biology. Co-stimulatory receptors such as CD4 are most closely associated with activation of TCR signalling necessary for antigen-specific immune responses. It is generally believed that CD4 serves to bring LCK to the immune synapse to phosphorylate CD3 and stabilize TCR–pMHC binding^50^; however, CD4 is necessary to amplify, not necessarily initiate, TCR signalling^51^. Thus, the precise role of CD4 in peripheral T cell responses remains unclear. In light of our results, we propose that CD4 can signal directly through LCK and contribute to T cell function during immune cell interactions by independently activating CDK1 to modulate nuclear transport, in turn influencing gene expression and cell function. The presence of CD4 on other immune cells like macrophages and dendritic cells that do not express the TCR supports the idea that CD4 signalling may well drive discrete cellular responses to influence immune cell function. Greater understanding of CD4 and, by extension CD8, signalling in immune cells and the consequences thereof is expected to reveal exciting new biology, informing novel pathways for improved immunotherapy by harnessing the power of co-stimulatory signalling to unleash bespoke immune cell responses.

## Methods

### Cells

Peripheral blood mononuclear cells (PBMCs) were isolated from leukocyte cones from healthy donors (NHS Blood and Transplant Services) by density centrifugation using Lympho 24+ Spin medium (PluriSelect) and pluriMate II tubes (PluriSelect). PBMCs were cryopreserved in 10% DMSO (Sigma-Aldrich) in FCS (LabTech). CD4^+^ T cells were isolated by negative selection using the MojoSort Human CD4 T cell Isolation Kit (BioLegend) and cultured in RPMI1460 medium supplemented with 10% FCS, 1% penicillin–streptomycin and 1% GlutaMax (Thermo Fisher Scientific) (complete RPMI medium) with 10 IU ml^−1^ IL-2 (Center for AIDS Reagents (CFAR), National Institutes of Biological Standard and Control, UK). For CD25/CD69 depletions biotin-Ab cocktail from the MojoSort Human CD4 T cell isolation kit was supplemented with biotin–anti-CD25 (M-A251, BioLegend) and biotin–anti-CD69 (FN50, BioLegend) antibodies (both at 10 μg per 50 million cells) to retain CD25/CD69 expressing cells in the negative fraction. Purity was assessed by flow cytometry (described below). Isolated CD4 T cells (8–12 × 10^6^ cells) were activated in Nunclon Delta T25 flasks (Thermo Fisher Scientific) using plate-bound anti-CD3 antibody (OKT3, 5 μg per flask, BioLegend) and soluble anti-CD28 antibody (CD28.2, 2 μg ml^−1^, BioLegend). After 3 days of activation, cells were cultured/rested in fresh medium (without CD3/CD28 antibodies) for another 2 days before being used for infections or co-cultures. Resting purified CD4 T cells were cultured in presence of 10 IU ml^−1^ IL-2 without CD3/CD28 activation. Jurkat T cell lines clone E6-1 (ATCC TIB-152) and clone 1G5 (containing Tat-driven luciferase reporter, obtained from AIDS Research and Reference Reagent Program, Division of AIDS, NIAID, NIH) were cultured in complete RPMI medium. HEK293T/17 cells (ATCC, CRL-11268) and HeLa-TZMbl cells (CFAR) were cultured in DMEM GlutaMax medium (Thermo Fisher Scientific) supplemented with 10% FCS and 1% penicillin–streptomycin.

### Plasmids

pNL4.3 plasmid (donated by M. Martin) was from the CFAR. pNL4.3 ΔEnv and pNL4.3 ΔVpr was provided by R. Sloan^52^. pCH040 and pCH077 were provided by G. Towers and were originally obtained through the NIH AIDS Reagent Program from J. Kappes and C. Ochsenbauer. pNLENG1-IRES NL4.3 Env and pNLENG1-IRES YU2 Env were provided by D. Levy^53^. pHGFP-GFPCA was generated previously and provided by V. Pathak and encodes GFP fused to CA in the NL4.3 ΔEnv backbone^20^. Parental pHGFP plasmid contains WT CA sequence^20^. pHGFP-mStayGold-CA was generated by replacing GFP sequence from pHGFP-GFPCA plasmid between the MluI and XbaI restriction sites with the mStayGold sequence cloned from the pRSETB/mStayGold plasmid (Addgene, 212017) using the following primers 5′-TCAGTGACGCGTATGGTGTCTACAGGCGAGGAGC and 5′-TCAGTCTCTAGACAGGTGGGCCTCCAGGGTC. pHGFP-Spot-tag-CA was generated by replacing the GFP sequence from the pHGFP-GFPCA plasmid between the MluI and XbaI restriction sites with the following primers also digested with MluI and XbaI enzymes: 5′-CGCGTCCAGACCGCGTGCGCGCCGTGAGCCATTGGAGCAGCGGCGGAT and 5′-CTAGATCCGCCGCTGCTCCAATGGCTCACGGCGCGCACGCGGTCTGGA. pBLAM-Vpr was obtained from Addgene and pAdVantage from Promega. pMDG expressing VSV glycoprotein was from Genscript. pWEAU_d15_410_5017 expressing HIV-1 WEAU isolate Env and pFurin were provided by L. McCoy. pCAT001 expressing codon-optimized HIV-1 HXB2 Env was obtained from Addgene (171061) NL4.3 Env-F522Y described previously^17^ was generated by site-directed mutagenesis using the QuickChange Lightning kit (Agilent) and the following primers: F522Y forward: 5′-GCTTTGTTCCTTGGGTAT TTGGGAGCAGCAGG; and F522Y reverse: 5′-CCTGCTGCTCCCAAA TACCCAAGGAACAAAGC.

### Viruses

Viruses were made by transfecting 293T cells (7 × 10^6^ cells seeded per T175 flask) with 10 μg HIV-1 plasmid using Fugene6 (Promega) in 1:3 ratio. HIV-1 NL4.3 ΔEnv and Env-F522Y virions were pseudotyped with Env from the primary isolate transmitter/founder virus WEAU by co-transfecting with pWEAU Env (5 μg) and pFurin (2.5 μg) plasmids. VSVg-pseudotyped NL4.3 ΔEnv and Env-F522Y were co-transfected with pMDG (2.5 μg). Blam-Vpr virus was made by co-transfecting pNL4.3 ΔVpr (10 μg), pBLAM-Vpr (2.5 μg) and pAdVantage (2 μg). GFP–CA virus was made by co-transfecting pNL4.3 WT (10 μg) with pHGFP-GFPCA (1 μg) as described previously^20^. mStayGold-CA virus was made by co-transfecting pHGFP (NL4.3 ΔEnv, 12 μg) with pHGFP-mStayGold-CA (1 μg) and pCAT001 HXB2 Env (1 μg) and pFurin (2.5 μg). Spot-CA virus was made by co-transfecting pHGFP (NL4.3 ΔEnv, 12 μg) with pHGFP-Spot-tag-CA (1 μg) and pCAT001 HXB2 Env (1 μg) and pFurin (2.5 μg). Viral supernatants were collected 48 h and 72 h after transfection, filtered through 0.45-μm syringe-driven filter, purified and concentrated by ultracentrifugation through 20% sucrose cushion and resuspended in complete RPMI medium. For experiments involving quantitative PCR (qPCR) analysis of viral DNA, the product supernatants were treated with DNase I (Sigma-Aldrich) for 1 h at 37 °C before ultracentrifugation. Viral titres were measured by quantifying supernatant RT activity by SG-PERT assay^54^. Alternatively, and where indicated, quantification of infectivity (TCID_50_ per ml) was determined by titration of viral supernatants on HeLa-TZMbl reporter cells using Luciferase Bright-Glo substrate (Promega) and GloMax luminometer (Promega).

### HIV-1 infections, VS priming and bead priming

#### Donor cell infection

Purified primary activated CD4 T cells (donors) were infected on day 5 after activation by spinoculation (2 h, 1,200*g*, 25 °C) with Env-pseudotyped NL4.3 Env-F522Y virus or NL4.3 ΔEnv virus (1.0 and 0.5 U RT per 10^6^ cells, respectively) in the presence of DEAE-dextran (0.1 μg per 10^6^ cells). Jurkat donor cells were infected with VSV-g pseudotyped NL4.3 Env-F522Y and NL4.3 ΔEnv virus (0.5U RT per 10^6^ cells) by gravity infection for 4 h before changing the medium. Infected donor cells were incubated for 2 days before use, and infection was confirmed by flow cytometry.

#### VS priming

Target T cells (resting or activated) were prelabelled with 2.5 μM eFluor 450 cell proliferation dye (Thermo Fisher Scientific) according to the manufacturer’s instructions. Dye-labelled resting target T cells were then co-cultured with autologous primary donor T cells at a 1:1 ratio for 24 h and challenged with NL4.3 WT, NL4.3 GFP–CA, CH040, CH077 (1 U RT per 10^6^ cells unless otherwise indicated) or CXCR4- and CCR5-tropic GFP reporter viruses (2 U RT per 10^6^ cells). Infection was measured 72 h later (unless otherwise indicated) by flow cytometry staining for cell surface CD4 and intracellular viral Gag (described below). Alternatively, for activated target T cell experiments, primary target T cells were used at day 5 after activation and co-cultured with either HIV-1 infected autologous activated donor cells or Jurkat donor cells. After 24 h, VS-primed targets were challenged with second virus as above, and infection was measured 24 h later.

#### Bead priming

Polystyrene streptavidin-coated beads with 5 μm diameter (Bangs Laboratories) were coated with biotin-tagged anti-CD4 antibody (OKT4, BioLegend), biotin-tagged anti-CD3 antibody (OKT3, BioLegend) or biotin tagged BG505 SOSIP Env (gift from L. McCoy) according to the manufacturer’s instructions. For 25% bead binding capacity (equivalent to 7 × 10^5^ biotin molecules per bead), 5 × 10^6^ beads were coated with 1 μg anti-CD4 or anti-CD3 antibody or 2.5 μg SOSIP Env. Unless otherwise indicated, beads were coated at 25% binding capacity. Streptavidin-coated nanobeads with 130 nm diameter (BioLegend) were coated in excess anti-CD4 antibody or SOSIP Env (25 μg ml^−1^) to achieve 100% binding capacity. Target T cells (resting or activated) were incubated with beads for 1 h at 1:1 ratio (unless otherwise indicated) before infection as described above.

#### CCS assay

Activated primary donor cells were infected with WT NL4.3 virus (0.5 U RT per 10^6^ cells) for 2 days as described above. Resting target cells were labelled with eFluor 450 Cell Proliferation Dye as described above and mixed with infected donor cells in 1:1 ratio (total 2 × 10^5^ cells per well). For cell-free infection comparison, 2 × 10^5^ target cells per well were infected with WT NL4.3 (2 U RT per 10^6^ cells), which resulted in equal target cell infection levels at 6 h.p.i., compared to CCS 6 h after coculture as measured by flow cytometry and immunofluorescence (described below).

For qPCR analysis, approximately 5 × 10^5^ target cells were sorted from co-cultures as described below. For inhibitor experiments, resting target T cells were either untreated or pretreated with LCK and CDK1/2 signalling pathway inhibitors and mixed with donor cells to allow for virus CCS. After 8 h and 24 h of coculture, neutralizing anti-Env antibody (VRC01, 20 μg ml^−1^, CFAR) and fusion inhibitor (Enfuvirtide [T20] 0.1 μM, CFAR) were added to block further HIV-1 CCS and infection of target T cells was measured after 72 h of co-culture by flow cytometry.

#### Jurkat 1G5 CCS assay

Jurkat T cells (WT, E6-1 clone) were HIV-1 infected as described above and co-cultured in a 1:1 ratio with uninfected 1G5 Jurkat target T cells. Luciferase expression was measured after 24 h of coculture using Luciferase Bright-Glo substrate and GloMax luminometer.

### Blam-Vpr assay

Cells were infected with NL4.3 Blam-Vpr virus (1 U RT per 10^6^ cells) for 4 h and β-lactamase activity was measured using the LiveBLAzer CCF2-AM Kit (Thermo Fisher Scientific) as described previously^55^.

### Inhibitors

Inhibitors were added to cells as indicated, either 1 h before co-cultures or 1 h before adding virus and used at the following concentrations. EFV (HIV RT inhibitor, 5 μM), maraviroc (CCR5 antagonist, 0.2 μM), AMD3100 (CXCR4 antagonist, 0.5 μM) and raltegravir (HIV integrase inhibitor, 5 μM) were purchased from CFAR. AB1010 (LCK inhibitor, 2 μM), IPA-3 (PAK1 inhibitor, 5 μM) and THZ1 (CDK7 inhibitor, 0.05 μM) were purchased from ApexBio. U0126 (ERK inhibitor, 4 μM), SP 600125 (JNK inhibitor, 5 μM), AKT INH VIII (AKT inhibitor, 0.1 μM), sotrastaurin (PKC inhibitor, 4 μM), MLN8237 (Aurora A inhibitor, 0.25 μM), GSK461364 (PLK1 inhibitor, 0.8 μM), NSC 663284 (CDC25 inhibitor, 4 μM), BMS-265246 (CDK1/2 inhibitor, 2 μM), palbociclib (CDK4/6 inhibitor, 1 μM) and eltanexor (exportin-1 inhibitor, 0.5 μM) were purchased from Cayman Chemicals.

### T cell signalling

To measure T cell signalling during VS-priming, donor and target T cells were stained with Zombie Aqua Live/Dead dye (BioLegend) and chilled on ice before being mixed and incubated on ice for 20 min to allow for donor–target conjugate formation. Similarly, bead and target T cells were incubated on ice to allow for bead–target conjugate formation. Co-cultures were then incubated in a 37 °C water bath for 0–25 min, before being fixed with warm fixation buffer (4% formaldehyde in PBS) to arrest signalling. Signalling activation was measured by flow cytometry using phospho-specific antibodies (described below).

### Flow cytometry

Cells were washed in PBS and stained with Zombie Aqua or UV Live/Dead dye (1:500, BioLegend) and antibodies against surface markers (detailed below) in PBS for 15 min at room temperature. Cells were then washed in PBS and fixed in 4% formaldehyde in PBS for 30 min. When only intracellular Gag expression was analysed, cells were permeabilized in intracellular staining permeabilization wash buffer (BioLegend) for 10 min and stained with anti-Gag antibody (KC57, PE (1:350) or FITC (1:150), Beckman Coulter) for 20 min and washed. When co-staining with other intracellular markers, the cells were permeabilized with 0.25% Triton X-100 (Sigma-Aldrich) in PBS for 15 min, blocked with 3% BSA (Sigma-Aldrich) and 0.1% Triton X-100 in PBS for 45 min, washed in PBS and stained in antibody diluted in 1% BSA in PBS for 30 min. Unconjugated primary antibodies were detected using the following secondary antibodies: anti-rabbit IgG (Poly4064, BV421 or PE, 1:200) or anti-mouse IgG (Poly4053, PE or Alexa647, 1:200) from BioLegend. For phospho-CDK1 Tyr15 staining, cells were first stained phospho-CDK1 Tyr15 followed by anti-mouse secondary antibody. Cells were then washed and incubated with additional phospho-CDK1 Tyr15 to block any excess secondary antibody binding sites and then surface stained with fluorophore-conjugated mouse anti-CD3, anti-CD4 and anti-CD8.

The following surface antigen antibodies were used: CD3 (UCHT1, FITC (1:100) or BV711 (1:250), BioLegend), CD4 (RPA-T4, APC (1:200) or APC-Fire750 (1:100), BioLegend) CD8 (SK1, BV605, BioLegend, 1:250), CD45RO (UCHL1, PerCP-Cy5.5, BioLegend, 1:150), CD69 (FN50, APC-Fire750, BioLegend, 1:100), CD25 (M-A251, PE-Dazzle594, BioLegend, 1:200), CD38 (HIT2, BV510, BioLegend, 1:150), HLA-DR (L243, BV785, BioLegend, 1:150), PD-1 (EH12.2H7, PE, BioLegend, 1:200), CD98 (REA387, PE-Vio770, Miltenyi Biotec, 1:150).

The following intracellular antigen antibodies were used: phospho-LCK Tyr394 (A18002D, PE, BioLegend, 1:150), phospho-LCK Tyr505 (REA673, APC, Miltenyi Biotec, 1:150), phospho-Zap70 Tyr319 (1503310, Pe-Cy7, BioLegend, 1:150), phospho-Zap70 Tyr292 (REA41, PE, Miltenyi Biotec, 1:150), phospho-ERK Thr202/Tyr204 (6B8B69, Alexa647, BioLegend, 1:150), phospho-AKT Ser473 (M89-61, PE-CF594, BD, 1:150), phospho-CDK1 Thr14 (A20004B, Alexa647, BioLegend, 1:150), phospho-CDK1 Tyr15 (A21009D, BioLegend, 1:200), phospho-CDK1 Thr161 (9114, Cell Signaling Technology, 1:200), total CDK1 (9112, Cell Signaling Technology, 1:200; or 2A11E4, CoraLite Plus 647, Proteintech, 1:250) phospho-Wee1 Ser642 (D47G5, Cell Signaling Technology, 1:200), total Wee (D10D2, Cell Signaling Technology, 1:200), Ki-67 (PE, BioLegend, 1:150), cyclin B1 (V152, Alexa647, BioLegend, 1:150), Glut1 (EPR3915, Abcam, 1:600), RRM1 (D12F12, Cell Signaling Technology, 1:250), RRM2 (E7Y9J, Cell Signaling Technology, 1:250), RRM2B (EPR8816, Abcam, 1:300) and HA tag (16B12, APC, BioLegend, 1:150). For EdU incorporation, cells were pulse labelled with 5 μM EdU for 3 h before Live/Dead stain and fixation. Cells were permeabilized and incorporated EdU was click-labelled with Cy3 dye using the EdU Cy3 Imaging Kit (ApexBio) according to the manufacturer’s instructions. After click-labelling, cells were washed in PBS and stained for surface antigens (CD3, CD4, CD8) as described above. Cell proliferation was measured by staining cells with 5 μM eFluor450 cell proliferation dye and assessing the decrease in eFluor450 fluorescence after 72 h compared with unstimulated cells. All of the samples were analysed on the BD Fortessa or BD Fortessa X20 (BD) system using BD FACSDiva Software v.9.0 (BD) and analysed by FlowJo v.10 (BD).

### Live-cell sorting

To isolate donor and target T cells after co-culture by flow sorting, donor cells were prelabelled with CellTrace Far Red dye (Thermo Fisher Scientific) and target T cells prelabelled with eFluor450 Cell Proliferation Dye. Resting target T cells were co-cultured with activated donor cells for 24 h to allow for VS-priming and then infected for 24 h as described above. Cells were then stained with Zombie Green Live/Dead dye and passed through 20 μm cell strainer before sorting for the live target T cell population on MACSQuant Tyto (Miltenyi Biotec) cell sorter using high-speed cartridges. The purity of sorted target T cell populations was confirmed to be >99% pure by flow cytometry analysis. Sorted resting target T cells were then either returned to culture or collected immediately for analysis. Alternatively, resting or activated target T cells were co-cultured with activated donor cells for 1 h or 24 h as indicated and sorted before infection.

### *CDK1* mRNA electroporation

Active (T14F/Y15F) and inactive (T14F/Y15F/T161A) CDK1 mutant sequences were HA-tagged at the N-terminus, codon optimized and synthesized as gBlocks (IDT) with following sequences: active: 5′-TATCCTTACGACGTGCCCGACTACGCAGGGGGCAGTGGCGGTATGGAGGAC TATACGAAGATCGAGAAAATTGGAGAGGGCGCTTTCGGCGTTGTATACAAAGGGAGACACAAGACTACAGGACAGGTG GTAGCAATGAAGAAAATTCGCCTTGAATCTGAGGAAGAGGGGGTACCCTCCACGGCGATTCGGGAAATCTCACTCCTCAAAGAACTGCGACACCCAAATATCGTATCACTTCAGGACGTTCTCATGCAAGATAGTAGACTGTACCTCATATTCGAGTTCTTGTCAATGGATTTGAAAAAATATCTTGATAGTATCCCACCAGGCCAATACATGGACAGTTCCCTGGTGAAGTCTTATCTCTACCAGATTCTGCAAGGTATCGTCTTTTGCCACTCCCGGAGGGTATTGCACAGAGACCTCAAGCCCCAAAACCTTCTGATTGACGACAAGGGGACCATAAAGTTGGCGGACTTTGGCCTGGCTAGGGCATTTGGTATACCAATTCGGGTTTATACCCACGAAGTAGTAACACTGTGGTACAGGAGCCCCGAGGTCCTGCTCGGCTCAGCGAGGTATTCAACCCCGGTGGACATCTGGAGCATTGGCACTATTTTTGCAGAACTCGCAACTAAGAAGCCTCTTTTTCACGGCGATTCCGAAATTGATCAACTCTTCCGAATTTTTCGGGCATTGGGCACGCCTAACAACGAGGTCTGGCCGGAGGTGGAGAGTTTGCAGGATTACAAGAATACGTTTCCGAAATGGAAACCTGGCAGCCTTGCCAGCCATGTGAAGAACCTCGATGAAAATGGCCTTGACCTCTTGTCCAAGATGCTTATCTACGACCCAGCGAAACGAATCAGCGGGAAGATGGCCCTGAACCATCCCTACTTCAATGACCTGGACAACCAGATCAAGAAGATGTGA; inactive 5′-TATCCTTACGACGTGCCCGACTACGCAGGGGGCAGTGGCGGTATGGAGGACTATACGAAGATCGAGAAAATTGGAGAGGGCGCTTTCGGCGTTGTATACAAAGGGAGACACAAGACTACAGG ACAGGTGGTAGCAATGAAGAAAATTCGCCTTGAATCTGAGGAAGAGGGGGTACCCTCCACGGCGATTCGGGAAATCTCACTCCTCAAAGAACTGCGACACCCAAATATCGTATCACTTCAGGACGTTCTCATGCAAGATAGTAGACTGTACCTCATATTCGAGTTCTTGTCAATGGATTTGAAAAAATATCTTGATAGTATCCCACCAGGCCAATACATGGACAGTTCCCTGGTGAAGTCTTATCTCTACCAGATTCTGCAAGGTATCGTCTTTTGCCACTCCCGGAGGGTATTGCACAGAGACCTCAAGCCCCAAAACCTTCTGATTGACGACAAGGGGACCATAAAGTTGGCGGACTTTGGCCTGGCTAGGGCATTTGGTATACCAATTCGGGTTTATGCACACGAAGTAGTAACACTGTGGTACAGGAGCCCCGAGGTCCTGCTCGGCTCAGCGAGGTATTCAACCCCGGTGGACATCTGGAGCATTGGCACTATTTTTGCAGAACTCGCAACTAAGAAGCCTCTTTTTCACGGCGATTCCGAAATTGATCAACTCTTCCGAATTTTTCGGGCATTGGGCACGCCTAACAACGAGGTCTGGCCGGAGGTGGAGAGTTTGCAGGATTACAAGAATACGTTTCCGAAATGGAAACCTGGCAGCCTTGCCAGCCATGTGAAGAACCTCGATGAAAATGGCCTTGACCTCTTGTCCAAGATGCTTATCTACGACCCAGCGAAACGAATCAGCGGGAAGATGGCCCTGAACCATCCCTACTTCAATGACCTGGACAACCAGATCAAGAAGATGTG.

The pCDNA3.1 vector was linearized by PCR using following primers: 5′-TGACCTGGACAACCAGATCAAGAAGATGTGAGGGCCCGTTTAAACCCGCTGATC and 5′-CCCTGCGTAGTCGGGCACGTCGTAAGGATACATGGTGGCCATGCTAGCCAGC. *CDK1* sequences were then inserted using the HiFi DNA Assembly kit (NEB). For in vitro transcription, *CDK1* vectors were first linearized by PCR using following primers: 5′-CATGGTGATGCGGTTTTGGCAGTACATCAATGG and 5′-CAGAAGCCATAGAGCCCACCGCATCC. RNA was then transcribed, capped and polyadenylated using the HiScribe T7 ARCA mRNA Kit with tailing (NEB) according to manufacturer’s instructions and purified using the Monarch RNA cleanup kit (NEB). Resting CD4 T cells were electroporated with mRNA using the Neon Transfection System (Thermo Fisher Scientific). In brief, 1.5 × 10^6^ cells were resuspended in 100 μl buffer T with 0–6 μg mRNA per 10^6^ cells and electroporated with 1 pulse at 2,200 V for 20 ms. Cells were infected or collected for immunofluorescence analysis (described below) at 8 h after electroporation and CDK1 expression was monitored by flow cytometry using HA antibodies (described above).

### *CDK1* CRISPR–Cas9 knockout

The method for CRISPR–Cas9 knockouts in resting CD4 T cells was described previously^56^. In brief, lyophilized tracrRNA and crRNA (IDT) were resuspended at 160 μM in IDT Duplex buffer. Non-targeting control (NTC) and *CDK1*-targeting cRNAs contained GGGTTCCTAGTACTGCAATT and CCATACCCATTGACTAACTA sequences, respectively. Single-stranded donor oligonucleotides (ssODN, sequence: TTAGCTCTGTTTACGTCCCAGCGGGCATGAGAGTAACAAGAGGGTGTGGTAATATTACGGTACCGAGCACTATCGATACAATATGTGTCATACGGACACG, IDT) were also resuspended at 160 μM in IDT Duplex buffer. To generate Cas9–RNPs 1.5 μl of each tracrRNA and crRNA was mixed and incubated at 37 °C for 30 min. Then, 1 μl of ssODN was added and incubated at 37 °C for 5 min. Next, 5 μl of 20 μM EnGen Spy Cas9 HF1 (NEB) was added and incubated for further 15 min at 37 °C. Meanwhile, 1.5 × 10^6^ CD4 T cells were washed and resuspended in 95 μl buffer T, mixed with 9 μl Cas9-RNP solution and electroporated (1 pulse, 2,200 V, 20 ms) using the Neon transfection system. The knockout efficiency was monitored by measuring CDK1 expression using flow cytometry; it took 10–12 days after electroporation to detect decreased expression of CDK1.

### RT–qPCR analysis

After cell sorting, DNA was extracted from 6–8 × 10^5^ target T cells using DNeasy blood and tissue kit (Qiagen). Early RT and late RT products were quantified using primers for RU5 region (strong stop codon) and second-strand transfer, respectively^57^. 2-LTR circles were quantified using primers for the 2-LTR junction^58^. qPCR products were quantified using TaqMan Gene Expression Master Mix (Thermo Fisher Scientific) and the 7500 Real-Time PCR System (Applied Biosystems), using the following primers: RU5-forward: 5′-GCCTCAATAAAGCTTGCCTTGA; RU5-reverse: 5′-TGACTAAAAGGGTCTGAGGGATCT; RU5-probe: 5′-FAM-AGAGTCACACAACAGACG GGCACACACTA-TAMRA; second-strand transfer-forward: 5′-TAGTCAGTGTGGAAAATCTCTAGC; second-strand transfer-reverse: 5′-CTTCTAGCC TCCGCTAGTCAA; second-strand transfer-probe: 5′-FAM-TCGACGCAGGACTCGGCTTGCT-TAMRA; 2-LTR junction-forward: 5′-AACTAGAGATCCCTCAGACCCTTTT; 2-LTR junction-reverse: 5′-CTTGTCTTCGTTGGGAGTGAATT; 2-LTR junction-probe: 5′-FAM-CTAGAGATTTTCCACACTGAC-TAMRA.

HIV proviral integration was quantified from DNA extracts using the Alu-Gag PCR method as described previously^16,59^.

### Quantification of cellular dNTPs

dNTPs were extracted from 8 × 10^5^ cells per sample and quantified by qPCR using the single-nucleotide incorporation and PCR extension method as described previously^60^. To measure the effect of increasing intracellular dNTP levels, cells were incubated with exogenous deoxynucleosides (2 mM each of deoxyadenosine, deoxythymidine, deoxyguanosine and deoxycytidine; Thermo Fisher Scientific) as described previously^61^ for 24 h before HIV-1 infection.

### Immunoblotting

For whole-cell lysates, cells (1.5 × 10^6^ cells per sample) were washed in PBS and lysed in RIPA buffer supplemented with benzonase. Isolation of nuclei was performed as described previously^62^. In brief, 1.5 × 10^6^ cells per sample were lysed in buffer A (320 mM sucrose, 10 mM HEPES, 8 mM MgCl_2_, 1× Roche EDTA-free cOmplete Protease Inhibitor) with 0.1% (v/v) Triton X-100 and nuclei were isolated by centrifugation. The nuclear fraction was washed twice in buffer A without Triton X-100 before lysis in RIPA buffer. Cell/nuclear lysates were separated by SDS–PAGE, transferred onto a nitrocellulose membrane and blocked in 5% skimmed milk, 0.05% Tween-20 in PBS or TRIS-buffered saline (for phospho-SAMHD1 detection) for 1 h. The blots were then probed with following primary antibodies overnight at 4 °C: TPR (PA5-54048, Invitrogen, 1:2,000), Nup153 (ab96462, Abcam, 1:2,000), Nup98 (C39A3, Cell Signaling Technology, 1:5,000), Nup62 (610497, BD), Nup54 (16232-1-AP, Proteintech, 1:5,000), Lamin A/C (ab108595, Abcam, 1:2,000) GAPDH (W17079A, BioLegend, 1:2,000), phospho-SAMHD1 Thr592 (D702M, Cell Signaling Technology, 1:2,000), total SMAHD1 (W19081C, BioLegend, 1:2,000) and tubulin (DM1A, Sigma-Aldrich, 1:2,000). After washing, blots were incubated with the following fluorescently tagged secondary antibodies for 1 h at room temperature (1:10,000): anti-rabbit IgG (RDye 800CW, Abcam), anti-rat IgG (Alexa680, Abcam) and anti-mouse IgG (IRDye 680RD, Abcam). Immunoblots were imaged using the Odyssey DXl Infrared Imager (LI-COR Biosciences) and analysed with Image Studio Lite software v5.2 (LI-COR Biosciences).

### iSIM imaging

Resting primary T cells were VS-primed or bead primed as described above, infected with GFP–CA NL4.3 virus (1 U RT per 10^6^ cells) for 6 h or 24 h and then transferred to an optical 96-well µ-plate (10^5^ cells per well) (Ibidi) coated with poly-D-lysine (Thermo Fisher Scientific) for 2 h before being fixed with methanol-free 4% formaldehyde (Thermo Fisher Scientific) and washed in PBS. For KPNΒ1 localization assays, cells were treated with either DMSO or exportin-1 inhibitor for 3 h before fixation. For permeabilization, cells were incubated for 15 min with 0.25% Triton X-100 in PBS followed by washing in PBS. Cells were then blocked for 45 min at 4 °C with 3% BSA and 0.1% Triton X-100 in PBS followed by washing in PBS. Cells were incubated with primary antibody (1:100) overnight in 1% BSA in PBS at 4 °C, washed in PBS and incubated with secondary antibody (1:500) in 1% BSA in PBS for 1 h at 4 °C. The following primary antibodies were used: lamin A/C (ab108595, Abcam; sc-7292, Santa Cruz Biotechnology), KPNΒ1 (ab2811, Abcam), Nup358 (ab64276, Abcam), Nup214 (Abcam, ab70497), hCG1 (Abcam, ab192609), Nup54 (Proteintech, 16232-1-AP), Nup58 (Atlas Antibodies, HPA039360), Nup62 (SC48389, Santa Cruz Biotechnology), Nup98 (C39A3, Cell Signaling Technology), Nup153 (ab96462, Abcam), Nup50 (A301-783A, Fortis), TPR (ab58344, Abcam; PA5-54048, Invitrogen) and HIV-1 CA (ab309159, Abcam). The following secondary antibodies were used: anti-rabbit IgG (Alexa568, A-11011, Invitrogen) and anti-mouse IgG (Alexa568, A-11004, Invitrogen). Nuclear staining was carried out by incubating cells with NucSpot650 (Biotium) for 60 min at room temperature.

iSIM imaging was performed using a VT-iSIM imaging system (Visitech). Four-channel images (405 nm/488 nm/561 nm/640 nm) of the medial T cell plane were acquired using a CoolLED pE-4000 MultiLazer, Hamamatsu Orca camera and a Plan Apo ×60/1.4 NA oil objective with ×1.5 zoom. iSIM post-processing was applied in NIS-Elements (Nikon), using a five-iteration Richardson–Lucy deconvolution algorithm providing an effective *xy* resolution of 150–170 nm.

### Live-cell iSIM imaging

Cells were incubated at 37 °C for 30 mins in Leibovits-L15 (no Phenol Red, Thermo Fisher Scientific) with 10% FCS with 1× CellBrite-Steady-550 (30107-T, Biotium) membrane stain and 1× NucSpot-650 (40082, Biotium) live nuclear stain. Cells were washed once in PBS and resuspended in Leibovits-L15 (no Phenol Red, Thermo Fisher Scientific) with 10% FCS, with uncoated 5-μm-diameter (Bangs Laboratories) biotin tagged BG505 SOSIP Env and seeded in an Ibidi µ-slide glass-bottom 18-well chamber (81817-900, Ibidi) precoated with poly-d-lysine (Thermo Fisher Scientific). Cells were transferred to environmental chamber (37 °C, 5% CO^2^) of the VT-iSIM imaging system (Visitech) and incubated for 1 h. The cells were then challenged with 2 U RT per 10^6^ cells of HIV-1-StayGold-CA virus and imaging carried out immediately. Live-cell imaging was performed using the VT-iSIM imaging system (Visitech). Three-channel (488 nm/561 nm/640 nm), 21 slice (increment = 0.5 μm) *z* stacks were acquired using the CoolLED pE-4000 MultiLazer, Hamamatsu Orca camera and a Plan Apo ×100/1.45 NA oil objective. Continuous imaging was carried out for 4 h with acquisition intervals of 2 mins. In both conditions, cells were preselected for evident perinuclear HIV-1-StayGold virion localizations and cropped to single-cell fields of view for post-processing. iSIM post-processing was applied in NIS-Elements (Nikon), using a five-iteration Richardson–Lucy deconvolution algorithm providing an effective *xy* resolution of 130 nm.

### iSIM image analysis

Resting target T cells were manually identified as cells labelled with eFluor450 Cell Proliferation Dye and the target population was collected by cropping a bounding box to the target T cell mask. Cropped target T cell populations were processed for quantitative analysis in Fiji imageJ^63^ using ImageJ macros (https://github.com/MattVXWhelan/Mesner_Whelan_et_al/blob/main/ImagejMacros). HIV-1 GFP–CA^+^ target T cells were manually scored for GFP–CA cellular localization using Fiji ImageJ’s plot profile function. GFP–CA^+^ puncta were classed as NE associated by colocalization of 50% of the puncta intensity histogram with perinuclear lamin A/C or TPR. Puncta were classified as nuclear by colocalization with NucSpot650 nuclear signal. Surface/cytosolic GFP puncta were determined as the remainder of the T-cell-associated GFP–CA^+^ puncta. Automated image analysis of Nuclear/NPC TPR and Nup62 was carried out using Cellprofiler 4^64^. T cell nuclei were segmented from a mask generated from Nucspot650. Nucleoporin puncta were segmented and nucleoporin intensity was quantified within the nuclear mask which encompassed peripheral NPCs. Cell profiler pipelines are available online (https://github.com/MattVXWhelan/Mesner_Whelan_et_al/blob/main/CellProfiler_Analysis). Single-NPC quantification was carried out using the Fiji ImageJ analysis plot profile function to measure the pixel intensity of HIV-CA-associated Nup62/TPR-positive NPCs. For live-cell image analysis, pre-processing steps were applied in Fiji ImageJ. This included *xyz* drift correction using NucSpot650 channel as a guide using Fast4Dreg^65^, followed by ImageJ macros applying a 50 px rolling-ball background subtraction followed by autothreshold of channel intensities for visualization. HIV-SG puncta were identified through fitting of a Gaussian function (Fiji ImageJ, Thunderstorm plugin) and manually tracked in 3D. Perinuclear localization was visually scored by localization of HIV-1-StayGold full width at half maximum (FWHM) to the adjacent NucSpot650 signal. Nuclear localization was determined by peak intensity colocalization of HIV-1-StayGold virions with NucSpot650 Intensity. Live-cell ImageJ macros used in analysis are available online (https://github.com/MattVXWhelan/Mesner_Whelan_et_al/blob/main/LiveCellPipeline).

### dSTORM imaging

The samples were prepared and analysed using the ONI Discovery kit (Oxford Nanoimaging). Nucleoporins were labelled with anti-FG-Nups antibody (MAb414, ab24609, Abcam, 1:100) and anti-Nup54 (16232-1-AP, Proteintech, 1:100) or anti-lamin A/C (ab108595, Abcam, 1:100). Primary labelled Nups were labelled with CF568-cojugated secondary antibodies (Biotium, 1:500) and Spot-tagged CA virus with Spot-Label Alexa Fluor 647 nanobody (Proteintech, 1:200). dSTORM imaging was carried out on the ONI Nanoimager (Oxford Nanoimaging) in the presence of ONI BCubed buffer. After bead *xy* channel calibration, two-channel images were acquired on HiLO or TIRF mode at 30 ms for 10,000 frames. Localization renderings were generated in NimOS software (Oxford Nanoimaging). Nanoimager results filtering based on the manufacturer’s recommendations: photon count (>300), localization precision: *xy* (5–25 nm), sigma *xy* (50–250 nm). For visualization, localizations were also rendered in Fiji ImageJ using the ThunderSTORM plugin^66^.

### dSTORM analysis

NPC HIV-1 interactions were quantified using a similar approach to that previously described^25^. For translocation analysis, localization frequencies were quantified using the NimOS line Histogram tool (width: 3 px, bins: 40). Diameter calculations were carried out by a Gaussian fit of localization frequency distributions followed by a FWHM calculation for each histogram (Prism GraphPad). For Nup54 localization measurements. A 2D Gaussian fitting of localizations was carried out in Napari (v.0.5)^67^. The analysis pipeline is available online (https://github.com/MattVXWhelan/Mesner_Whelan_et_al/blob/main/STORM_Analysis).

### MS analysis

#### Sample preparation

Resting CD4^+^ T cells (1–1.2 × 10^7^ cells per condition, 4 independent PBMC donors) were pretreated with CDK1/2 inhibitor (BMS-265246, 2 μM, Cayman Chemicals) or DMSO control for 1 h and stimulated with beads (anti-CD4, Env, anti-CD3/CD28) for 6 h. Cells were collected by centrifugation and washed twice in PBS. Cell pellets were boiled in lysis buffer (5% SDS, 5 mm tris(2-carboxyethyl)phosphine, 10 mm chloroacetamide, 100 mm Tris, pH 8.5) for 10 min followed by micro-tip probe sonication (Q705 Sonicator from Fisherbrand) for 2 min with pulses of 1 s on and 1 s off at 50% amplitude. The protein concentration was estimated using the BCA assay (Thermo Fisher Scientific). Protein digestion was automated on the KingFisher APEX robot (Thermo Fisher Scientific) in 96-well format using a protocol described previously^68^ with minor modifications. The 96-well comb is stored in plate 1, the sample in plate 2 in a final concentration of 70% acetonitrile with magnetic MagReSyn Hydroxyl beads (ReSyn Biosciences) in a protein/bead ratio of 1:2. Wash solutions are in plates 3–5 (95% acetonitrile) and plates 6–7 (70% ethanol). Plate 8 contained 300 μl digestion solution of 100 mm Tris pH 8.5 and trypsin (Promega) in an enzyme:protein ratio of 1:100. The protein aggregation was carried out in two steps of 1 min mixing at medium mixing speed, followed by a 10 min pause each. The sequential washes were performed in 2.5 min and slow speed, without releasing the beads from the magnet. The digestion was set to 16 h at 37 °C with slow speed. Protease activity was quenched by acidification with trifluoroacetic acid (TFA) to a final pH of 2 and the resulting peptide mixture was purified on the OASIS HLB 96-well plate (Waters). Purified peptides were dried in a Savant DNA120 (Thermo Fisher Scientific) system and 5% of the 200 µg total amount was separated and used directly for liquid chromatography (LC)–MS for proteomics analysis, while the rest was used for phospho-enrichment.

#### Phosphopeptide enrichment

Phosphopeptide enrichment was performed on the KingFisher APEX robot (with the protocol described previously^68^) using the MagReSyn Zr-IMAC HP beads (ReSyn Biosciences). The robot layout was as follows: plate 1: the 96-well comb; plate 2: 40 µl of Zr-IMAC HP beads dissolved in 160 µl of the loading buffer (80% acetonitrile, 5% TFA and 0.1 M glycolic acid); plate 3: 500 µl of loading buffer; plate 4: and samples dissolved in 200 µl of loading buffer. Plates 5–7 were filled with 500 μl wash solutions: plate 5 contained the loading buffer, plate 6 contained 80% ACN (acetonitrile) with 1% TFA and plate 7 contained 10% ACN and 0.2% TFA. Plate 8 contained 200 μl 1% ammonia for elution. The beads were washed in loading buffer for 5 min at medium mixing speed, followed by binding of the phosphopeptides for 20 min at medium speed. The sequential washes were performed for 2 min at fast speed. Phosphopeptides were eluted in 10 min at medium mixing speed. Eluted phosphopeptides were acidified with 10% TFA to pH < 3 and purified in an OASIS HLB 96-well plate (Waters).

#### LC–MS

Dried peptides and phosphopeptides were dissolved in 0.5% TFA analysed using the Ultimate3000 high-performance liquid chromatography system coupled online to an Orbitrap Eclipse mass spectrometer (Thermo Fisher Scientific). Buffer A consisted of water acidified with 0.1% formic acid, while buffer B was 80% acetonitrile and 20% water with 0.1% formic acid. The peptides were first trapped for 1 min at 30 μl min^−1^ with 100% buffer A on a trap (0.3 mm by 5 mm with PepMap C18, 5 μm, 100 Å; Thermo Fisher Scientific); after trapping, the peptides were separated by a 50 cm µPAC Neo HPLC column (Thermo Fisher Scientific). The gradient was 3–35% B in 48 min at 750 nl min^−1^. Buffer B was then raised to 55% in 3 min and increased to 99% for the cleaning step. Peptides were ionized using a spray voltage of 2 kV and a capillary heated at 275 °C. The mass spectrometer was set to acquire full-scan MS spectra (350 to 1400 mass/charge ratio) at a mass resolution of 120,000 and an automated gain control (AGC) target value of 250% (RF lens of 40% and maximum injection time of 45 ms). For MS/MS fragmentation, we chose the DIA approach, but we used different settings for normal or phosphopeptides: for normal proteomics, the *m*/*z* range used was 361–1,033 *m*/*z* divided in 56 windows (12 *m*/*z* each with 1 Da overlap) with an AGC of 1,000% and resolution of 15,000. For phosphopeptides, we used 28 windows of 24 Th each with an overlap of 1 (*m*/*z* range from 472–1,143) and a resolution of 30,000. All raw files were transformed in .htrms format and analysed by Spectronaut v.18.5 with direct DIA analysis. We used the library generated automatically using Human reference proteome (20,420 sequences downloaded from UniProt) together with the MaxQuant contaminants list and standard settings: for normal peptides we used the BGS factory settings and, for the phospho files, we used the BGS phospho PTM workflow.

#### Data analysis

Abundance proteomics data were analysed using R (v.4.1.1). Batch correction was performed using ComBat from the sva package (v.3.42.0) to account for donor-specific effects. No imputation was performed and proteins that were not detected in at least two donors across at least 60% of experimental conditions were excluded. Differential expression analysis was performed using the limma package (v.3.50.1) with donor as a blocking factor. Benjamini–Hochberg correction was applied. Phosphoproteome data were analysed using Perseus v.2.1.6.0^69^. Phosphosites that were undetected in two or more donors per condition were excluded and the remaining missing values were imputed from normal distribution for each sample to calculate fold changes and statistical significance. The MS proteomics data have been deposited to the ProteomeXchange Consortium via the PRIDE partner repository with the dataset identifier PXD062217.

### Statistical analysis

Statistical tests were performed using Prism v.10 (GraphPad) and details of statistical tests used are indicated. Statistical tests for proteomics data were performed using R and Perseus as described above.

### Reporting summary

Further information on research design is available in the Nature Portfolio Reporting Summary linked to this article.

## Online content

Any methods, additional references, Nature Portfolio reporting summaries, source data, extended data, supplementary information, acknowledgements, peer review information; details of author contributions and competing interests; and statements of data and code availability are available at 10.1038/s41586-026-10453-3.

## Supplementary information

Supplementary Fig. 1 Uncropped immunoblots from Extended Data figures. **a**, Immunoblots from Extended Data Fig. 4d, showing SAMHD1 phosphorylation in VS-primed and CD3/CD28 activated T cells. **b**, Immunoblots from Extended data Fig. 8 showing nucleoporin, lamin and GAPDH levels in total cell and nuclear extracts of bead-primed T cells. The red rectangles show cropped areas. MW, molecular weight; EB, empty bead.

Reporting Summary

Supplementary Table 1 Processed total proteome abundance MS data. The table shows all the peptides identified in the MS experiment. The first tab of the spreadsheet shows the sample legend and the second tab shows the raw data. These data were used to calculate changes in expression of NPC components shown in Fig. 5f.

Supplementary Table 2 Processed phosphoproteome MS data. The table shows all the phosphopeptides identified in the MS experiment. The first tab of the spreadsheet shows the sample legend, the second tab shows the raw data and the third tab shows the imputed data. These data were used to calculate changes in phosphorylation of NPC components shown in Fig. 5g.

Supplementary Video 1 Live-cell imaging of HIV-1 virions in empty-bead-treated resting T cells. Cells were labelled with nuclear label (magenta), preincubated for 1 h with empty beads and challenged with HIV-1 mStayGold-CA virions (green). Cells were imaged continuously every 2 min for 4 h. The white circle identifies a tracked virion that shows cytoplasmic association at 56 min, docks at nuclear rim at 2 h 6 min and disassociates at 2 h 34 min, demonstrating unsuccessful nuclear import.

Supplementary Video 2 Live-cell imaging of HIV-1 virions in Env-bead-stimulated resting T cells. Cells were labelled with nuclear label (magenta), preincubated for 1 h with Env-beads and challenged with HIV-1 mStayGold-CA virions (green). Cells were imaged continuously every 2 min for 4 h. The white circle identifies a tracked virion that shows cytoplasmic association at 24 min, docking at 54 min and clear nuclear import at 2 h 24 min.

## Source data

Source Data Fig. 1

Source Data Fig. 2

Source Data Fig. 3

Source Data Fig. 4

Source Data Fig. 5

Source Data Extended Data Fig. 1

Source Data Extended Data Fig. 3

Source Data Extended Data Fig. 4

Source Data Extended Data Fig. 5

Source Data Extended Data Fig. 6

Source Data Extended Data Fig. 7

Source Data Extended Data Fig. 8

## Data Availability

The MS proteomics data have been deposited to the ProteomeXchange Consortium via the PRIDE partner repository under dataset identifier PXD062217. Source data are provided with this paper.

## Code availability

The microscopy analysis pipeline code is available at GitHub (https://github.com/MattVXWhelan/Mesner_Whelan_et_al/blob/main).

## References

[1] Li, Q. et al. Peak SIV replication in resting memory CD4^+^ T cells depletes gut lamina propria CD4^+^ T cells. *Nature* **434**, 1148–1152 (2005). 15793562 10.1038/nature03513

[2] Zhang, Z. et al. Sexual transmission and propagation of SIV and HIV in resting and activated CD4^+^ T cells. *Science* **286**, 1353–1357 (1999). 10558989 10.1126/science.286.5443.1353

[3] Eckstein, D. A. et al. HIV-1 actively replicates in naive CD4^+^ T T cells residing within human lymphoid tissues. *Immunity* **15**, 671–682 (2001). 11672548 10.1016/s1074-7613(01)00217-5

[4] Nishimura, Y. et al. Resting naive CD4^+^ T cells are massively infected and eliminated by X4-tropic simian-human immunodeficiency viruses in macaques. *Proc. Natl Acad. Sci. USA* **102**, 8000–8005 (2005). 15911767 10.1073/pnas.0503233102 PMC1142395

[5] Stevenson, M., Stanwick, T. L., Dempsey, M. P. & Lamonica, C. A. HIV-1 replication is controlled at the level of T cell activation and proviral integration. *EMBO J.* **9**, 1551–1560 (1990). 2184033 10.1002/j.1460-2075.1990.tb08274.x PMC551849

[6] Swiggard, W. J. et al. Human immunodeficiency virus type 1 can establish latent infection in resting CD4^+^ T cells in the absence of activating stimuli. *J. Virol.* **79**, 14179–14188 (2005). 16254353 10.1128/JVI.79.22.14179-14188.2005 PMC1280214

[7] Zack, J. A. et al. HIV-1 entry into quiescent primary lymphocytes: molecular analysis reveals a labile, latent viral structure. *Cell* **61**, 213–222 (1990). 2331748 10.1016/0092-8674(90)90802-l

[8] Shan, L. et al. Transcriptional reprogramming during effector-to-memory transition renders CD4^+^ T cells permissive for latent HIV-1 infection. *Immunity* **47**, 766–775 (2017). 29045905 10.1016/j.immuni.2017.09.014 PMC5948104

[9] Wilen, C. B., Tilton, J. C. & Doms, R. W. HIV: cell binding and entry. *Cold Spring Harb. Perspect. Med.* **2**, a006866 (2012). 10.1101/cshperspect.a006866 PMC3405824 22908191

[10] Mattei, S., Glass, B., Hagen, W. J., Krausslich, H. G. & Briggs, J. A. The structure and flexibility of conical HIV-1 capsids determined within intact virions. *Science* **354**, 1434–1437 (2016). 27980210 10.1126/science.aah4972

[11] Beck, M. & Hurt, E. The nuclear pore complex: understanding its function through structural insight. *Nat. Rev. Mol. Cell Biol.* **18**, 73–89 (2017). 27999437 10.1038/nrm.2016.147

[12] Guttinger, S., Laurell, E. & Kutay, U. Orchestrating nuclear envelope disassembly and reassembly during mitosis. *Nat. Rev. Mol. Cell Biol.* **10**, 178–191 (2009). 19234477 10.1038/nrm2641

[13] Jolly, C., Kashefi, K., Hollinshead, M. & Sattentau, Q. J. HIV-1 cell to cell transfer across an Env-induced, actin-dependent synapse. *J. Exp. Med.* **199**, 283–293 (2004). 14734528 10.1084/jem.20030648 PMC2211771

[14] Chen, P., Hubner, W., Spinelli, M. A. & Chen, B. K. Predominant mode of human immunodeficiency virus transfer between T cells is mediated by sustained Env-dependent neutralization-resistant virological synapses. *J. Virol.* **81**, 12582–12595 (2007). 17728240 10.1128/JVI.00381-07 PMC2169007

[15] Sourisseau, M., Sol-Foulon, N., Porrot, F., Blanchet, F. & Schwartz, O. Inefficient human immunodeficiency virus replication in mobile lymphocytes. *J. Virol.* **81**, 1000–1012 (2007). 17079292 10.1128/JVI.01629-06 PMC1797449

[16] Reuschl, A. K. et al. HIV-1 Vpr drives a tissue residency-like phenotype during selective infection of resting memory T cells. *Cell Rep.* **39**, 110650 (2022). 35417711 10.1016/j.celrep.2022.110650 PMC9350556

[17] Bergeron, L., Sullivan, N. & Sodroski, J. Target cell-specific determinants of membrane fusion within the human immunodeficiency virus type 1 gp120 third variable region and gp41 amino terminus. *J. Virol.* **66**, 2389–2397 (1992). 1548769 10.1128/jvi.66.4.2389-2397.1992 PMC289034

[18] Brenchley, J. M. et al. CD4^+^ T cell depletion during all stages of HIV disease occurs predominantly in the gastrointestinal tract. *J. Exp. Med.* **200**, 749–759 (2004). 15365096 10.1084/jem.20040874 PMC2211962

[19] Chomont, N. et al. HIV reservoir size and persistence are driven by T cell survival and homeostatic proliferation. *Nat. Med.* **15**, 893–900 (2009). 19543283 10.1038/nm.1972 PMC2859814

[20] Burdick, R. C. et al. HIV-1 uncoats in the nucleus near sites of integration. *Proc. Natl Acad. Sci. USA* **117**, 5486–5493 (2020). 32094182 10.1073/pnas.1920631117 PMC7071919

[21] Burdick, R. C. et al. HIV-1 uncoating requires long double-stranded reverse transcription products. *Sci. Adv.* **10**, eadn7033 (2024). 38657061 10.1126/sciadv.adn7033 PMC11042746

[22] Dharan, A., Bachmann, N., Talley, S., Zwikelmaier, V. & Campbell, E. M. Nuclear pore blockade reveals that HIV-1 completes reverse transcription and uncoating in the nucleus. *Nat. Microbiol.* **5**, 1088–1095 (2020). 32483230 10.1038/s41564-020-0735-8 PMC9286700

[23] Muller, T. G., Zila, V., Muller, B. & Krausslich, H. G. Nuclear capsid uncoating and reverse transcription of HIV-1. *Annu. Rev. Virol.* **9**, 261–284 (2022). 35704745 10.1146/annurev-virology-020922-110929

[24] Vasiliver-Shamis, G., Cho, M. W., Hioe, C. E. & Dustin, M. L. Human immunodeficiency virus type 1 envelope gp120-induced partial T-cell receptor signaling creates an F-actin-depleted zone in the virological synapse. *J. Virol.* **83**, 11341–11355 (2009). 19710135 10.1128/JVI.01440-09 PMC2772796

[25] Kreysing, J. P. et al. Passage of the HIV capsid cracks the nuclear pore. *Cell* **188**, 930–943 (2025). 39826544 10.1016/j.cell.2024.12.008

[26] Burdick, R. C. et al. Dynamics and regulation of nuclear import and nuclear movements of HIV-1 complexes. *PLoS Pathog.* **13**, e1006570 (2017). 28827840 10.1371/journal.ppat.1006570 PMC5578721

[27] Blethrow, J. D., Glavy, J. S., Morgan, D. O. & Shokat, K. M. Covalent capture of kinase-specific phosphopeptides reveals Cdk1-cyclin B substrates. *Proc. Natl Acad. Sci. USA* **105**, 1442–1447 (2008). 18234856 10.1073/pnas.0708966105 PMC2234163

[28] Matthews, H. K., Bertoli, C. & de Bruin, R. A. M. Cell cycle control in cancer. *Nat. Rev. Mol. Cell Biol.* **23**, 74–88 (2022). 34508254 10.1038/s41580-021-00404-3

[29] Massacci, G., Perfetto, L. & Sacco, F. The cyclin-dependent kinase 1: more than a cell cycle regulator. *Br. J. Cancer* **129**, 1707–1716 (2023). 37898722 10.1038/s41416-023-02468-8 PMC10667339

[30] Jang, S. & Engelman, A. N. Capsid-host interactions for HIV-1 ingress. *Microbiol. Mol. Biol. Rev.* **87**, e0004822 (2023). 37750702 10.1128/mmbr.00048-22 PMC10732038

[31] Dickson, C. F. et al. The HIV capsid mimics karyopherin engagement of FG-nucleoporins. *Nature* **626**, 836–842 (2024). 38267582 10.1038/s41586-023-06969-7 PMC10881392

[32] Fu, L. et al. HIV-1 capsids enter the FG phase of nuclear pores like a transport receptor. *Nature* **626**, 843–851 (2024). 38267583 10.1038/s41586-023-06966-w PMC10881386

[33] Otsuka, S. et al. A quantitative map of nuclear pore assembly reveals two distinct mechanisms. *Nature* **613**, 575–581 (2023). 36599981 10.1038/s41586-022-05528-w PMC9849139

[34] Siliciano, J. D. & Siliciano, R. F. In vivo dynamics of the latent reservoir for HIV-1: new insights and implications for cure. *Annu. Rev. Pathol.* **17**, 271–294 (2022). 34736342 10.1146/annurev-pathol-050520-112001

[35] Agosto, L. M., Herring, M. B., Mothes, W. & Henderson, A. J. HIV-1-infected CD4^+^ T cells facilitate latent infection of resting CD4^+^ T cells through cell-cell contact. *Cell Rep.* **24**, 2088–2100 (2018). 30134170 10.1016/j.celrep.2018.07.079

[36] Murooka, T. T. et al. HIV-infected T cells are migratory vehicles for viral dissemination. *Nature* **490**, 283–287 (2012). 22854780 10.1038/nature11398 PMC3470742

[37] Unutmaz, D., KewalRamani, V. N., Marmon, S. & Littman, D. R. Cytokine signals are sufficient for HIV-1 infection of resting human T lymphocytes. *J. Exp. Med.* **189**, 1735–1746 (1999). 10359577 10.1084/jem.189.11.1735 PMC2193071

[38] Sattentau, Q. Avoiding the void: cell-to-cell spread of human viruses. *Nat. Rev. Microbiol.* **6**, 815–826 (2008). 18923409 10.1038/nrmicro1972

[39] Zimmerli, C. E. et al. Nuclear pores dilate and constrict in cellulo. *Science* **374**, eabd9776 (2021). 34762489 10.1126/science.abd9776

[40] Cicala, C. et al. HIV envelope induces a cascade of cell signals in non-proliferating target cells that favour virus replication. *Proc. Natl Acad. Sci. USA* **99**, 9380–9385 (2002). 12089333 10.1073/pnas.142287999 PMC123149

[41] Wojcechowskyj, J. A. et al. Quantitative phosphoproteomics reveals extensive cellular reprogramming during HIV-1 entry. *Cell Host Microbe* **13**, 613–623 (2013). 23684312 10.1016/j.chom.2013.04.011 PMC4104530

[42] Uhlmann, F., Bouchoux, C. & Lopez-Aviles, S. A quantitative model for cyclin-dependent kinase control of the cell cycle: revisited. *Philos. Trans. R. Soc. Lond. B* **366**, 3572–3583 (2011). 22084384 10.1098/rstb.2011.0082 PMC3203462

[43] Maeshima, K., Iino, H., Hihara, S. & Imamoto, N. Nuclear size, nuclear pore number and cell cycle. *Nucleus* **2**, 113–118 (2011). 21738834 10.4161/nucl.2.2.15446 PMC3127093

[44] Shen, Q. et al. The capsid lattice engages a bipartite NUP153 motif to mediate nuclear entry of HIV-1 cores. *Proc. Natl Acad. Sci. USA* **120**, e2202815120 (2023). 36943880 10.1073/pnas.2202815120 PMC10068764

[45] Ori, A. et al. Cell type-specific nuclear pores: a case in point for context-dependent stoichiometry of molecular machines. *Mol. Syst. Biol.* **9**, 648 (2013). 23511206 10.1038/msb.2013.4 PMC3619942

[46] Kuhn, T. M. & Capelson, M. Nuclear pore proteins in regulation of chromatin state. *Cells* **8**, 1414 (2019). 10.3390/cells8111414 PMC6912232 31717499

[47] Brady, T. et al. HIV integration site distributions in resting and activated CD4^+^ T cells infected in culture. *AIDS* **23**, 1461–1471 (2009). 19550285 10.1097/QAD.0b013e32832caf28 PMC2862484

[48] Chin, C. R. et al. Direct visualization of HIV-1 replication intermediates shows that capsid and CPSF6 modulate HIV-1 intra-nuclear invasion and integration. *Cell Rep.* **13**, 1717–1731 (2015). 26586435 10.1016/j.celrep.2015.10.036 PMC5026322

[49] Achuthan, V. et al. Capsid-CPSF6 interaction licenses nuclear HIV-1 trafficking to sites of viral DNA integration. *Cell Host Microbe* **24**, 392–404 (2018). 30173955 10.1016/j.chom.2018.08.002 PMC6368089

[50] Morch, A. M., Balint, S., Santos, A. M., Davis, S. J. & Dustin, M. L. Coreceptors and TCR signaling—the strong and the weak of it. *Front. Cell Dev. Biol.* **8**, 597627 (2020). 33178706 10.3389/fcell.2020.597627 PMC7596257

[51] Xu, H. & Littman, D. R. A kinase-independent function of Lck in potentiating antigen-specific T cell activation. *Cell* **74**, 633–643 (1993). 8358792 10.1016/0092-8674(93)90511-n

[52] Sloan, R. D. et al. Transcription of preintegrated HIV-1 cDNA modulates cell surface expression of major histocompatibility complex class I via Nef. *J. Virol.* **85**, 2828–2836 (2011). 21209113 10.1128/JVI.01854-10 PMC3067938

[53] Trinite, B. et al. Suppression of Foxo1 activity and down-modulation of CD62L (L-selectin) in HIV-1 infected resting CD4 T cells. *PLoS ONE* **9**, e110719 (2014). 25330112 10.1371/journal.pone.0110719 PMC4199762

[54] Pizzato, M. et al. A one-step SYBR Green I-based product-enhanced reverse transcriptase assay for the quantitation of retroviruses in cell culture supernatants. *J. Virol. Methods* **156**, 1–7 (2009). 19022294 10.1016/j.jviromet.2008.10.012

[55] Cavrois, M., De Noronha, C. & Greene, W. C. A sensitive and specific enzyme-based assay detecting HIV-1 virion fusion in primary T lymphocytes. *Nat. Biotechnol.* **20**, 1151–1154 (2002). 12355096 10.1038/nbt745

[56] Rathore, U. et al. CRISPR-Cas9 screen of E3 ubiquitin ligases identifies TRAF2 and UHRF1 as regulators of HIV latency in primary human T cells. *mBio* **15**, e0222223 (2024). 38411080 10.1128/mbio.02222-23 PMC11005436

[57] Mbisa, J. L., Delviks-Frankenberry, K. A., Thomas, J. A., Gorelick, R. J. & Pathak, V. K. Real-time PCR analysis of HIV-1 replication post-entry events. *Methods Mol. Biol.* **485**, 55–72 (2009). 10.1007/978-1-59745-170-3_5 PMC6800109 19020818

[58] Apolonia, L. et al. Stable gene transfer to muscle using non-integrating lentiviral vectors. *Mol. Ther.* **15**, 1947–1954 (2007). 17700544 10.1038/sj.mt.6300281

[59] Liszewski, M. K., Yu, J. J. & O’Doherty, U. Detecting HIV-1 integration by repetitive-sampling Alu-gag PCR. *Methods* **47**, 254–260 (2009). 19195495 10.1016/j.ymeth.2009.01.002 PMC2862469

[60] Purhonen, J., Banerjee, R., McDonald, A. E., Fellman, V. & Kallijarvi, J. A sensitive assay for dNTPs based on long synthetic oligonucleotides, EvaGreen dye and inhibitor-resistant high-fidelity DNA polymerase. *Nucleic Acids Res.* **48**, e87 (2020). 32573728 10.1093/nar/gkaa516 PMC7470940

[61] Baldauf, H. M. et al. SAMHD1 restricts HIV-1 infection in resting CD4^+^ T cells. *Nat. Med.* **18**, 1682–1687 (2012). 22972397 10.1038/nm.2964 PMC3828732

[62] Podtschaske, M. et al. Digital NFATc2 activation per cell transforms graded T cell receptor activation into an all-or-none IL-2 expression. *PLoS ONE* **2**, e935 (2007). 17895976 10.1371/journal.pone.0000935 PMC1978524

[63] Schindelin, J. et al. Fiji: an open-source platform for biological-image analysis. *Nat. Methods* **9**, 676–682 (2012). 22743772 10.1038/nmeth.2019 PMC3855844

[64] Stirling, D. R. et al. CellProfiler 4: improvements in speed, utility and usability. *BMC Bioinform.* **22**, 433 (2021). 10.1186/s12859-021-04344-9 PMC8431850 34507520

[65] Pylvanainen, J. W. et al. Fast4DReg—fast registration of 4D microscopy datasets. *J. Cell Sci.* **136**, jcs260728 (2023). 10.1242/jcs.260728 PMC10022679 36727532

[66] Ovesny, M., Krizek, P., Borkovec, J., Svindrych, Z. & Hagen, G. M. ThunderSTORM: a comprehensive ImageJ plug-in for PALM and STORM data analysis and super-resolution imaging. *Bioinformatics* **30**, 2389–2390 (2014). 24771516 10.1093/bioinformatics/btu202 PMC4207427

[67] Selzer, G. J. et al. napari-imagej: ImageJ ecosystem access from napari. *Nat. Methods* **20**, 1443–1444 (2023). 37596471 10.1038/s41592-023-01990-0 PMC10591722

[68] Koenig, C. et al. Protocol for high-throughput semi-automated label-free- or TMT-based phosphoproteome profiling. *STAR Protoc.* **4**, 102536 (2023). 37659085 10.1016/j.xpro.2023.102536 PMC10491724

[69] Tyanova, S. et al. The Perseus computational platform for comprehensive analysis of (prote)omics data. *Nat. Methods* **13**, 731–740 (2016). 27348712 10.1038/nmeth.3901

