# Supplementary material for: HIV-1 signalling remodels nuclear pores to licence infection
Source: Nature. 2026 May 6;654(8120):1044–54. doi: 10.1038/s41586-026-10453-3 (PMC13293875; doi:10.1038/s41586-026-10453-3)
Supplement: Supplementary file 1 — Uncropped immunoblots from Extended Data figures. a, Immunoblots from Extended Data Fig. 4d, showing SAMHD1 phosphorylation in VS-primed and CD3/CD28 activated T cells. b, Immunoblots from Extended data Fig. 8 showing nucleoporin, lamin and GAPDH levels in total cell and nuclear extracts of bead-primed T cells. The red rectangles show cropped areas. MW, molecular weight; EB, empty bead. [file 41586_2026_10453_MOESM1_ESM.pdf]

nature portfolio

https://doi.org/10.1038/s41586-026-10453-3

Supplementary information

HIV-1 signalling remodels nuclear pores to licence infection

In the format provided by the authors and unedited

HIV-1 signalling remodels nuclear pores to licence infection

Dejan Mesner[1,2*], Matthew X.V. Whelan[1,2*], Maitreyi Shivkumar[1,3], Ann-Kathrin Reuschl[1,2], Riccardo Zenezini Chiozzi[4], Konstantinos Thalassinos[4,5], Robertus A M de Bruin[6] and Clare Jolly[1,2]

[1]Division of Infection and Immunity, University College London, London WC1E 6BT, United Kingdom
[2]Current address: Centre for Immunobiology and Infection, Blizard Institute, Faculty of Medicine and Dentistry, Queen Mary University of London, E1 2AT, United Kingdom
[3]Current address: Leicester School of Pharmacy, De Montfort University, Leicester LE1 9BH, United Kingdom
[4]Institute of Structural and Molecular Biology, Division of Biosciences, University College London, London WC1E 6BT, United Kingdom
[5]Institute of Structural and Molecular Biology, School of Natural Sciences, Birkbeck College, University of London, London WC1E 7HX, United Kingdom
[6]Laboratory for Molecular Cell Biology, University College London, London WC1E 6BT United Kingdom
* These authors contributed equally.

Corresponding author Clare Jolly clare.jolly@qmul.ac.uk

Supplementary information

This file contains Supplementary Figure 1 and legends for Supplementary Table 1 and 2.

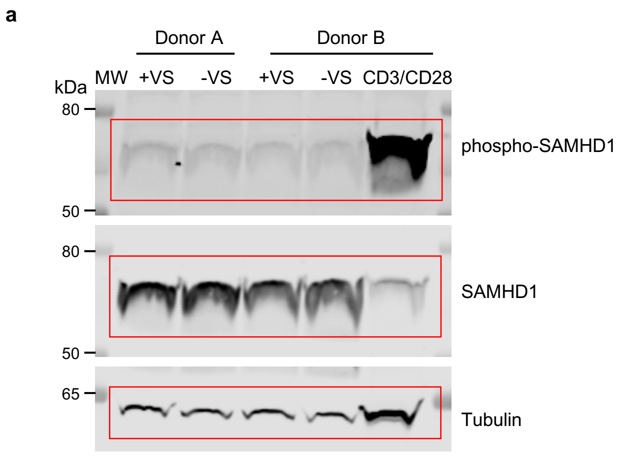

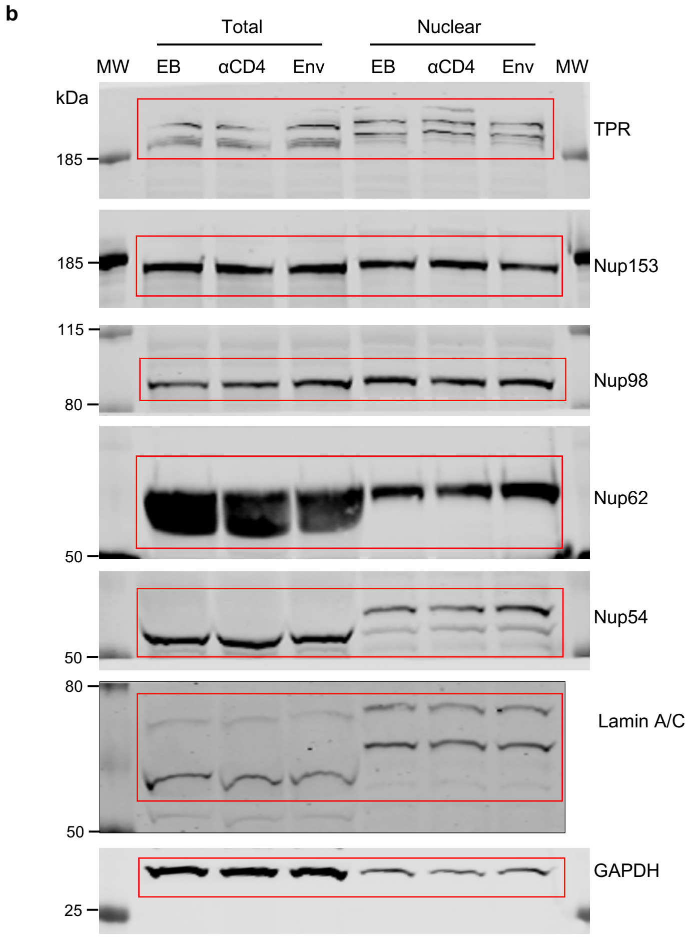

Supplementary Figure 1: Uncropped immunoblots from Extended Data figures.
(a) Immunoblots from Extended Data Figure 4d showing SAMHD1 phosphorylation in VS-primed and CD3/CD28 activated T-cells.
(b) Immunoblots from Extended data Figure 8 showing nucleoporin, lamin and GAPDH levels in total cell and nuclear extracts of bead-primed T-cells. The red rectangles show cropped areas. MW, molecular weight; EB, empty bead.

Supplementary Legends

Supplementary Table 1: Processed total proteome abundance mass spectrometry data
The table shows all the peptides identified in the mass spectrometry experiment. First tab of the spreadsheet shows the sample legend and the second tab shows the raw data. This data was used to calculate changes in expression of NPC components shown in Fig. 5f.

Supplementary Table 2: Processed phospho-proteome mass spectrometry data
The table shows all the phospho-peptides identified in the mass spectrometry experiment. First tab of the spreadsheet shows the sample legend, the second tab shows the raw data, and the third tab shows the imputed data. This data was used to calculate changes in phosphorylation of NPC components shown in Fig. 5g.
