# Supplementary material for: HIV-1 signalling remodels nuclear pores to licence infection
Source: Nature. 2026 May 6;654(8120):1044–54. doi: 10.1038/s41586-026-10453-3 (PMC13293875; doi:10.1038/s41586-026-10453-3)
Supplement: Supplementary file 2 — Reporting Summary [file 41586_2026_10453_MOESM2_ESM.pdf]

nature portfolio

Corresponding author(s): Clare Jolly
Last updated by author(s): Dec 17, 2025

# Reporting Summary

Nature Portfolio wishes to improve the reproducibility of the work that we publish. This form provides structure for consistency and transparency in reporting. For further information on Nature Portfolio policies, see our Editorial Policies and the Editorial Policy Checklist.

## Statistics

For all statistical analyses, confirm that the following items are present in the figure legend, table legend, main text, or Methods section.

| n/a | Confirmed | |
|---|---|---|
| ☐ | ☒ | The exact sample size (*n*) for each experimental group/condition, given as a discrete number and unit of measurement |
| ☐ | ☒ | A statement on whether measurements were taken from distinct samples or whether the same sample was measured repeatedly |
| ☐ | ☒ | The statistical test(s) used AND whether they are one- or two-sided<br>*Only common tests should be described solely by name; describe more complex techniques in the Methods section.* |
| ☐ | ☒ | A description of all covariates tested |
| ☐ | ☒ | A description of any assumptions or corrections, such as tests of normality and adjustment for multiple comparisons |
| ☐ | ☒ | A full description of the statistical parameters including central tendency (e.g. means) or other basic estimates (e.g. regression coefficient) AND variation (e.g. standard deviation) or associated estimates of uncertainty (e.g. confidence intervals) |
| ☐ | ☒ | For null hypothesis testing, the test statistic (e.g. $F$, $t$, $r$) with confidence intervals, effect sizes, degrees of freedom and $P$ value noted<br>*Give P values as exact values whenever suitable.* |
| ☒ | ☐ | For Bayesian analysis, information on the choice of priors and Markov chain Monte Carlo settings |
| ☒ | ☐ | For hierarchical and complex designs, identification of the appropriate level for tests and full reporting of outcomes |
| ☒ | ☐ | Estimates of effect sizes (e.g. Cohen's *d*, Pearson's *r*), indicating how they were calculated |

*Our web collection on statistics for biologists contains articles on many of the points above.*

## Software and code

Policy information about availability of computer code

| | |
|---|---|
| Data collection | BD FACSDiva Software Verison 9.0 (BD), Odyssey Infrared Imager software Image Studio Lite V5.2 (LI-COR), 7500 Software (Applied Biosystems), Spectronaut v18.5.<br>Image acquisition: VT-iSIM imaging system (Visitech). Nikon TI (Nikon). CoolLED pE-4000 MultiLazer, Hamamatsu Orca camera and a Plan Apo 60X/1.4NA Oil objective with 1.5X zoom. iSIM post-processing using NIS-Elements (Nikon). dSTORM: ONI Nanoimager (Oxford Nanoimaging). |
| Data analysis | FlowJo v10.10.0 (BD), 7500 Software (Applied Biosystems), Image Studio Lite V5.2 (LI-COR); GraphPad Prism10 Version 10.3.1, Jupyterlab 3.2.1 (https://github.com/jupyterlab/jupyterlab), Fiji image processing package (Fiji.sc), Cellprofiler V4.2.5 (Cellprofiler.org). NimOS software (Oxford Nanoimaging), ThunderSTORM ImageJ plugin (https://zitmen.github.io/thunderstorm).<br>Proteomics analysis: R (version 4.1.1), ComBat (sva package, version 3.42.0), Limma package (version 3.50.1), Perseus software (Cox Lab). |

For manuscripts utilizing custom algorithms or software that are central to the research but not yet described in published literature, software must be made available to editors and reviewers. We strongly encourage code deposition in a community repository (e.g. GitHub). See the Nature Portfolio guidelines for submitting code & software for further information.

## Data

Policy information about availability of data

All manuscripts must include a data availability statement. This statement should provide the following information, where applicable:

- Accession codes, unique identifiers, or web links for publicly available datasets
- A description of any restrictions on data availability
- For clinical datasets or third party data, please ensure that the statement adheres to our policy

Mass spectrometry raw data is deposited to the ProteomeXchange Consortium via the PRIDE partner repository with the dataset identifier PXD062217.

## Research involving human participants, their data, or biological material

Policy information about studies with human participants or human data. See also policy information about sex, gender (identity/presentation), and sexual orientation and race, ethnicity and racism.

| | |
|---|---|
| Reporting on sex and gender | Not applicable |
| Reporting on race, ethnicity, or other socially relevant groupings | Not applicable |
| Population characteristics | Not applicable |
| Recruitment | Not applicable |
| Ethics oversight | Not applicable |

Note that full information on the approval of the study protocol must also be provided in the manuscript.

# Field-specific reporting

Please select the one below that is the best fit for your research. If you are not sure, read the appropriate sections before making your selection.

☒ Life sciences ☐ Behavioural & social sciences ☐ Ecological, evolutionary & environmental sciences

For a reference copy of the document with all sections, see nature.com/documents/nr-reporting-summary-flat.pdf

# Life sciences study design

All studies must disclose on these points even when the disclosure is negative.

| | |
|---|---|
| Sample size | No statistical methods were used to pre-determine sample sizes. Multiple independent experiments were repeated to allow for appropriate statistical analysis. |
| Data exclusions | No data were excluded. |
| Replication | Experiments were performed independently a minimum of 3 times (unless otherwise stated) to allow for appropriate confidence. All attempts at replication were successful. |
| Randomization | No randomisation was performed. Experimental groups were treated identical except for the specific variable being tested, thus randomisation is not required. |
| Blinding | Blinding was not necessary all measurements were quantified by automated machines, and no data were excluded. |

# Reporting for specific materials, systems and methods

We require information from authors about some types of materials, experimental systems and methods used in many studies. Here, indicate whether each material, system or method listed is relevant to your study. If you are not sure if a list item applies to your research, read the appropriate section before selecting a response.

## Materials & experimental systems

| n/a | Involved in the study |
|---|---|
| | ☒ Antibodies |
| | ☒ Eukaryotic cell lines |
| ☒ | Palaeontology and archaeology |
| ☒ | Animals and other organisms |
| ☒ | Clinical data |
| ☒ | Dual use research of concern |
| ☒ | Plants |

## Methods

| n/a | Involved in the study |
|---|---|
| ☒ | ChIP-seq |
| | ☒ Flow cytometry |
| ☒ | MRI-based neuroimaging |

# Antibodies

Antibodies used

Antibody information listed follows the format: target (clone, conjugated fluorophore, manufacturer, catalogue number)

Antibodies used for flow cytometry:
anti-CD3 (UCHT1, FITC or BV711, Biolegend, 300406 or 300464)
anti-CD4 (RPA-T4, APC or APC-Fire750, Biolegend, 300514 or 300560)
anti-CD8 (SK1, BV605, Biolegend, 344742)
anti-CD45RO (UCHL1, PerCP-Cy5.5, Biolegend, 304222)
anti-CD69 (FN50, APC-Fire750, Biolegend, 310946)
anti-CD25 (M-A251, PE-Dazzle594, Biolegend, 356126)
anti-CD38 (HIT2, BV510, Biolegend, 303540)
anti-HLA-DR (L243, BV785, Biolegend, 307642)
anti-PD-1 (EH12.2H7, PE, Biolegend, 329906)
anti-CD98 (REA387, PE-Vio770, Miltenyi Biotec, 130-126-170).
anti-HIV-1 Gag (KC57, PE or FITC, Beckman Coulter, 6604665 or 6604667)
anti-phospho-Lck Y394 (A18002D, PE, Biolegend, 933104),
anti-phospho-Lck Y505 (REA673, APC, Miltenyi Biotec, 130-110-285)
anti-phospho-Zap70 Y319 (1503310, PE-Cy7, Biolegend. 683708)
anti-phospho-Zap70 Y292 (REA411, PE, Miltenyi Biotec, 130-106-945)
anti-phospho-ERK T202/Y204 (6B8B69, Alexa647, Biolegend, 369504),
anti-phospho-AKT S473 (M89-61, PE-CF594, BD, 562465)
anti-phospho-CDK1 T14 (A20004B, Alexa647, Biolegend, 947406)
anti-phospho-CDK1 Y15 (A21009D, unconjugated, Biolegend, 613852)
anti-phospho-CDK1 T161 (9114, unconjugated, Cell Signaling Technology, 9114S)
anti-CDK1 (9112, unconjugated, Cell Signaling Technology, discontinued)
anti-CDK1 (2A11E4, CoraLite Plus 647, Proteintech, CL647-67575)
anti-phospho-Wee1 S642 (D47G5, unconjugated, Cell Signaling Technology, 4910S)
anti-Wee (D10D2, unconjugated, Cell Signaling Technology, 13084S)
anti-Ki67 (Ki-67, PE, Biolegend, 350504)
anti-Cyclin B1 (V152, Alexa647, Biolegend, 647906)
anti-Glut1 (EPR3915, Alexa647, Abcam, ab195020)
anti-RRM1 (D12F12, unconjugated, Cell Signaling Technology, 8637S)
anti-RRM2 (E7Y9J, unconjugated, Cell Signaling Technology, 65939S)
anti-RRM2B (EPR8816, unconjugated, Abcam, ab154194)
anti-HA.11 epitope tag (16B12, APC, Biolegend, 901523)
anti-rabbit IgG (Poly4064, BV421 or PE, Biolegend, 406410 or 406421)
anti-mouse IgG (Poly4053, PE or Alexa647, Biolegend, 405307 or 405322)
Antibodies used for immunoblotting:
anti-phospho-SAMHD1 T592 (D702M, unconjugated, Cell Signaling Technology, 89930S)
anti-SMAHD1 (W19081C, unconjugated, Biolegend, 941002)
anti-tubulin (DM1A, unconjugated, Sigma, CP06-100UG)
anti-Nup54 (polyclonal, Proteintech, 16232-1-AP)
anti-Nup62, (53/Nucleoporin p62, unconjugated, BD, 610497)
anti-Nup98 (C39A3, unconjugated, Cell Signaling Technology, 2598S)
anti-Nup153 (SA1, unconjugated, Abcam, ab96462)
anti-Tpr (polyclonal, unconjugated, Invitrogen, PA5-54048)
anti-Lamin A/C (EPR4100, unconjugated, Abcam, ab108595)
anti-GAPDH (W17079A, unconjugatyed, Biolegend, 607901)
anti-rabbit IgG (polyclonal, IRDye 800CW, Abcam, ab216773)
anti-rat IgG (polyclonal, Alexa680, Abcam, ab175778)
anti-mouse IgG (polyclonal, IRDye 680RD, Abcam, ab216776)
Antibodies used for immunofluorescence:
anti-Lamin A/C (EPR4100, unconjugated, Abcam, ab108595)
anti-Lamin A/C (636, unconjugated, Santa Cruz Biotechnology, sc-7292)
anti-KPNB1 (3E9, unconjugated, Abcam, ab2811)
anti-Nup358 (polyclonal, unconjugated, Abcam, ab64276)
anti-Nup62 (E-4, unconjugated, Santa Cruz Biotechnology, SC48389)
anti-Nup62, (53/Nucleoporin p62, unconjugated, BD, 610497)
anti-Nup98 (C39A3, unconjugated, Cell Signaling Technology, 2598S)

anti-Nup153 (SA1, unconjugated, Abcam, ab96462)
anti-Nup50 (polyclonal, unconjugated, Fortis, A301-783A)
anti-Tpr (EPR8982, unconjugated, Abcam, ab58344)
anti-Tpr (polyclonal, unconjugated, Invitrogen, PA5-54048)
anti-Nup214 (polyclonal, unconjugated, Abcam, ab70497)
anti-hCG1 (EPR16545, unconjugated, Abcam, ab192609)
anti-Nup54 (polyclonal, unconjugated, Proteintech, 16232-1-AP),
anti-Nup58 (polyclonal, unconjugated, Atlas Antibodies, HPA039360)
anti-FG-Nups (MAb414, unconjugated, Abcam, ab24609)
anti-Nup210 (polyclonal, unconjugated, Novus Biologicals, NB100-93336)
anti-HIV-1 capsid (RM2002, unconjugated, Abcam, ab309159)
anti-Spot-tag (Spot-Label, Alexa647, Proteintech, ebAF647)
anti-rabbit IgG (polyclonal, Alexa568, Invitrogen, A-11011)
anti-mouse IgG (polyclonal, Alexa568, Invitrogen, A-11004).
anti-rabbit IgG (polyclonal, CF568, Biotium, 20801)
anti-mouse IgG (polyclonal, CF568, Biotium, 20800)

Validation

Appropriate negative and positive controls were included in experiments to confirm expected staining patterns and the absence of or low non-specific binding of antibodies.
Antibody validation information can be found at the manufacturer’s website with the following links:

Flow cytometry:
anti-CD3: https://www.biolegend.com/en-us/products/fitc-anti-human-cd3-antibody-863
anti-CD4: https://www.biolegend.com/en-us/products/apc-anti-human-cd4-antibody-8
anti-CD8: https://www.biolegend.com/en-us/products/brilliant-violet-605-anti-human-cd8-antibody-12406
anti-CD45RO: https://www.biolegend.com/en-us/products/percp-cyanine5-5-anti-human-cd45ro-antibody-5604
anti-CD69: https://www.biolegend.com/en-us/products/apc-fire-750-anti-human-cd69-antibody-13233
anti-CD25: https://www.biolegend.com/en-us/products/pe-dazzle-594-anti-human-cd25-antibody-9782
anti-CD38: https://www.biolegend.com/en-us/products/brilliant-violet-510-anti-human-cd38-antibody-13157
anti-HLA-DR: https://www.biolegend.com/en-us/products/brilliant-violet-785-anti-human-hla-dr-antibody-7975
anti-PD-1: https://www.biolegend.com/en-us/products/pe-anti-human-cd279-pd-1-antibody-4412
anti-CD98: https://www.miltenyibiotec.com/GB-en/products/cd98-antibody-anti-human-reafinity-rea387.html#conjugate=pe-vio-770:size=100-tests-in-200-ul
anti-HIV-1 Gag: https://www.mybeckman.uk/reagents/coulter-flow-cytometry/antibodies-and-kits/single-color-antibodies/hiv-1-core-antigen/6604667
anti-phospho-Lck Y394: https://www.biolegend.com/en-us/products/pe-anti-lck-phospho-tyr394-antibody-18616
anti-phospho-Lck Y505: https://www.miltenyibiotec.com/GB-en/products/lck-py505-antibody-anti-human-reafinity-rea673.html#conjugate=apc:size=100-tests-in-1-ml
anti-phospho-Zap70 Y319: https://www.biolegend.com/en-us/products/pe-cyanine7-anti-zap70-phospho-tyr319-syk-phospho-tyr352-antibody-14441
anti-phospho-Zap70 Y292: https://www.miltenyibiotec.com/GB-en/products/zap70-py292-antibody-anti-human-reafinity-rea411.html#conjugate=pe:size=100-tests-in-1-ml
anti-phospho-ERK T202/Y204: https://www.biolegend.com/en-us/products/alexa-fluor-647-anti-erk1-2-phospho-thr202-tyr204-antibody-12924
anti-phospho-AKT S473: https://www.bdbiosciences.com/en-gb/products/reagents/flow-cytometry-reagents/research-reagents/single-color-antibodies-ruo/pe-cf594-mouse-anti-akt-ps473.562465
anti-phospho-CDK1 T14: https://www.biolegend.com/en-us/products/alexa-fluor-647-anti-human-cdc2-phospho-thr14-antibody-22898
anti-phospho-CDK1 Y15: https://www.biolegend.com/en-us/products/purified-anti-cdc2-phospho-tyr15-antibody-23049
anti-phospho-CDK1 T161: https://www.cellsignal.com/products/primary-antibodies/phospho-cdc2-thr161-antibody/9114
anti-CDK1: https://www.cellsignal.com/products/primary-antibodies/cdc2-antibody/9112
anti-CDK1: https://www.ptglab.com/products/CDK1-Antibody-CL647-67575.htm
anti-HA.11 tag: https://www.biolegend.com/en-us/products/apc-anti-ha-11-epitope-tag-antibody-15870
anti-phospho-Wee1 S642: https://www.cellsignal.com/products/primary-antibodies/phospho-wee1-ser642-d47g5-rabbit-mab/4910
anti-Wee: https://www.cellsignal.com/products/primary-antibodies/wee1-d10d2-rabbit-mab/13084
anti-Ki67: https://www.biolegend.com/en-us/products/pe-anti-human-ki-67-antibody-6968
anti-Cyclin B1: https://www.biolegend.com/en-us/products/alexa-fluor-647-anti-cyclin-b1-antibody-13727
anti-Glut1: https://www.abcam.com/en-us/products/primary-antibodies/alexa-fluor-647-glucose-transporter-glut1-antibody-epr3915-ab195020
anti-RRM1: https://www.cellsignal.com/products/primary-antibodies/rrm1-d12f12-xp-rabbit-mab/8637
anti-RRM2: https://www.cellsignal.com/products/primary-antibodies/rrm2-e7y9j-xp-rabbit-mab/65939
anti-RRM2B: https://www.abcam.com/en-us/products/primary-antibodies/p53r2-antibody-epr8816-ab154194:
anti-rabbit IgG: https://www.biolegend.com/en-us/products/pe-donkey-anti-rabbit-igg-minimal-x-reactivity-9751
anti-mouse IgG: https://www.biolegend.com/en-us/products/pe-goat-anti-mouse-igg-minimal-x-reactivity-1418

Immunoblotting:
anti-phospho-SAMHD1 T592: https://www.cellsignal.com/products/primary-antibodies/phospho-samhd1-thr592-d7o2m-rabbit-mab/89930
anti-SAMHD1: https://www.biolegend.com/en-us/products/purified-anti-samhd1-antibody-20231
anti-Lamin A/C: https://www.abcam.com/en-us/products/primary-antibodies/lamin-a-lamin-c-antibody-epr4100-nuclear-envelope-marker-ab108595
anti-Nup62: https://www.bdbiosciences.com/en-gb/products/reagents/microscopy-imaging-reagents/immunofluorescence-reagents/purified-mouse-anti-nucleoporin-p62.610497?tab=product_details
anti-Nup98: https://www.cellsignal.com/products/primary-antibodies/nup98-c39a3-rabbit-mab/2598
anti-Nup153: https://www.abcam.com/en-us/products/primary-antibodies/nup153-antibody-sa1-ab96462

anti-Tpr: https://www.thermofisher.com/antibody/product/TPR-Antibody-Polyclonal/PA5-54048
anti-Nup54: https://www.ptglab.com/products/NUP54-Antibody-16232-1-AP.htm
anti-tubulin: https://www.sigmaaldrich.com/GB/en/product/mm/cp06
anti-GAPDH: https://www.biolegend.com/en-us/products/purified-anti-gapdh-antibody-15747
anti-rabbit IgG: https://www.abcam.com/en-us/products/secondary-antibodies/goat-rabbit-igg-h-l-irdye-800cw-preadsorbed-ab216773
anti-rat IgG: https://www.abcam.com/en-us/products/secondary-antibodies/goat-rat-igg-h-l-alexa-fluor-680-ab175778
anti-mouse IgG: https://www.abcam.com/en-us/products/secondary-antibodies/goat-mouse-igg-h-l-irdye-680rd-preadsorbed-ab216776

Immunofluorescence:
anti-Lamin A/C: https://www.abcam.com/en-us/products/primary-antibodies/lamin-a-lamin-c-antibody-epr4100-nuclear-envelope-marker-ab108595
anti-Lamin A/C: https://www.scbt.com/p/lamin-a-c-antibody-636?srsltid=AfmBOoqRkNB-CNWwYi7quC54NBBIhAES-uuEkmMACwqdou5iMLNnhzLH
anti-KPNB1: https://www.abcam.com/en-us/products/primary-antibodies/kpnb1-antibody-3e9-ab2811
anti-Nup358: https://www.abcam.com/en-us/products/primary-antibodies/ranbp2-antibody-ab64276
anti-Nup62: https://www.scbt.com/p/nucleoporin-p62-antibody-e-4?srsltid=AfmBOoq89czhjjp5YjuDm69gYjvMKii2jblaXRfKaY-J6kBM43ZmD_Fu
anti-Nup62: https://www.bdbiosciences.com/en-gb/products/reagents/microscopy-imaging-reagents/immunofluorescence-reagents/purified-mouse-anti-nucleoporin-p62.610497?tab=product_details
anti-Nup98: https://www.cellsignal.com/products/primary-antibodies/nup98-c39a3-rabbit-mab/2598
anti-Nup153: https://www.abcam.com/en-us/products/primary-antibodies/nup153-antibody-sa1-ab96462
anti-Nup50: https://www.fortislife.com/products/primary-antibodies/rabbit-anti-nup50-antibody/BETHYL-A301-783
anti-Tpr: https://www.abcam.com/en-us/products/primary-antibodies/tpr-antibody-epr8982-ab170940
anti-Tpr: https://www.thermofisher.com/antibody/product/TPR-Antibody-Polyclonal/PA5-54048
anti-Nup214: https://www.abcam.com/en-us/products/primary-antibodies/nup214-antibody-ab70497
anti-hCG1: https://www.abcam.com/en-us/products/primary-antibodies/nlp1-antibody-epr16545-ab192609
anti-Nup54: https://www.ptglab.com/products/NUP54-Antibody-16232-1-AP.htm
anti-Nup58: https://www.atlasantibodies.com/products/primary-antibodies/triple-a-polyclonals/anti-nup58-antibody-hpa039360/
anti-FG-Nup: https://www.abcam.com/en-us/products/primary-antibodies/nuclear-pore-complex-proteins-antibody-mab414-ab24609?srsltid=AfmBOorzHxzgbGX5kjQ3YTwXuQ9By4UZ8mZ0gQBVoS9CPT-mz0dc6-6H
anti-Nup210: https://www.novusbio.com/products/nup210-antibody_nb100-93336?srsltid=AfmBOoqx_Rn9fr2TsLOq8nqNBTz-L7AYoKsRFVXvj9lToJuUK1cKHB_x
anti-HIV-1 capsid: https://www.abcam.com/en-us/products/primary-antibodies/hiv1-p55-p24-p17-antibody-rm2002-ab309159
anti-Spot-tag: https://www.ptglab.com/products/Spot-Label-Alexa-Fluor-647-ebAF647.htm
anti-rabbit IgG: https://www.thermofisher.com/antibody/product/Goat-anti-Rabbit-IgG-H-L-Cross-Adsorbed-Secondary-Antibody-Polyclonal/A-11011
anti-mouse IgG: https://www.thermofisher.com/antibody/product/Goat-anti-Mouse-IgG-H-L-Cross-Adsorbed-Secondary-Antibody-Polyclonal/A-11004
anti-rabbit IgG: https://biotium.com/product/goat-anti-rabbit-igg-hl-highly-cross-absorbed-cf-dye-storm/
anti-mouse IgG: https://biotium.com/product/goat-anti-mouse-igg-hl-highly-cross-absorbed-cf-dye-storm/

## Eukaryotic cell lines

Policy information about cell lines and Sex and Gender in Research

| | |
|---|---|
| Cell line source(s) | Jurkat cell line clone E6-1 and HEK 293T/17 cells were obtained from the ATCC. Jurkat cell line clone 1G5 and HeLa-TZMbl cells were obtained from AIDS Research and Reference Reagent Program, Division of AIDS, NIH. |
| Authentication | Cell lines were commercially procured and confirmed to be the cell lines indicated and mycoplasma-free by the supplier. |
| Mycoplasma contamination | Random mycoplasma testing was conducted and cells tested negative. |
| Commonly misidentified lines (See ICLAC register) | None |

## Plants

| | |
|---|---|
| Seed stocks | *Report on the source of all seed stocks or other plant material used. If applicable, state the seed stock centre and catalogue number. If plant specimens were collected from the field, describe the collection location, date and sampling procedures.* |
| Novel plant genotypes | *Describe the methods by which all novel plant genotypes were produced. This includes those generated by transgenic approaches, gene editing, chemical/radiation-based mutagenesis and hybridization. For transgenic lines, describe the transformation method, the number of independent lines analyzed and the generation upon which experiments were performed. For gene-edited lines, describe the editor used, the endogenous sequence targeted for editing, the targeting guide RNA sequence (if applicable) and how the editor was applied.* |
| Authentication | *Describe any authentication procedures for each seed stock used or novel genotype generated. Describe any experiments used to assess the effect of a mutation and, where applicable, how potential secondary effects (e.g. second site T-DNA insertions, mosiacism, off-target gene editing) were examined.* |

# Flow Cytometry

## Plots

Confirm that:

☐ The axis labels state the marker and fluorochrome used (e.g. CD4-FITC).

☒ The axis scales are clearly visible. Include numbers along axes only for bottom left plot of group (a 'group' is an analysis of identical markers).

☒ All plots are contour plots with outliers or pseudocolor plots.

☒ A numerical value for number of cells or percentage (with statistics) is provided.

## Methodology

| | |
|---|---|
| Sample preparation | Peripheral blood mononuclear cells (PBMC) were isolated from leukocyte cones from healthy donors by density centrifugation using Lympho 24+ Spin medium and pluriMate II tubes (PluriSelect). PBMCs were cryopreserved in 10% DMSO in foetal calf serum, FCS. CD4 T-cells were isolated by negative selection using MojoSort Human CD4 T-cell Isolation Kit (Biolegend) and cultured in RPMI1460 medium supplemented with 10% FCS, 1% Pen-Strep and 1% GlutaMax (Thermo Fisher Scientific) with 10 IU/ml IL-2 (National Institutes of Biological Standard and Control).<br>For flow cytometry anlaysis cells were washed in PBS and stained with Zombie Aqua or UV Live/Dead dye (Biolegend) and antibodies against surface markers in PBS for 15min at room temperature. Cells were then washed in PBS and fixed in 4% formaldehyde in PBS for 30 min. When only intracellular Gag expression was analysed, cells were permeabilised in Intracellular Staining/Permeabilisation Wash Buffer (Biolegend) for 10 min and stained with anti-HIV-1 Gag antibody for 20 min and washed. When co-staining with other intracellular markers, the cells were permeabilised with 0.25% Triton-X100 (Sigma) in PBS for 15 min, blocked with 3% bovine serum albumin (BSA, Sigma) and 0.1 Triton-X100 in PBS for 45 min, washed in PBS and stained in antibody diluted in 1% BSA in PBS for 30min. |
| Instrument | All samples were analyzed on BD Fortessa or BD Fortessa X20. |
| Software | Data collection: FACSDiva Software v9.0 (BD). Data analysis: FlowJo v10.10.0 (BD). |
| Cell population abundance | Target cell purity after FACS sorting was in all samples >99.0%. |
| Gating strategy | Target cells were identified based on gating as lymphocytes (FSC vs SSC gate), target cells (Target dye e450 positive), single cells (FSC-A vs FCA-H gate), live cells (Zombie dye negative) and lastly CD4 cells (CD3 positive and CD8 negative). Cells in this live, singlet CD4 target cell gate were used for further downstream analysis of infection (Gag vs CD4 gate) or expression of various cell surface or intracellular markers. |

☒ Tick this box to confirm that a figure exemplifying the gating strategy is provided in the Supplementary Information.
